# Comparative transcriptomic analysis reveals genes regulating the germination of morphophysiologically dormant *Paris polyphylla* seeds during a warm stratification

**DOI:** 10.1371/journal.pone.0212514

**Published:** 2019-02-21

**Authors:** Dengqun Liao, Juan Zhu, Menghuan Zhang, Xian’en Li, Peng Sun, Jianhe Wei, Jianjun Qi

**Affiliations:** Institute of Medicinal Plant Development, Chinese Academy of Medical Sciences and Peking Union Medical College, Beijing PR China; Youngstown State University, UNITED STATES

## Abstract

We previously analyzed the expression of genes associated with *Paris polyphylla* var. *yunnanensis* seed maturation and dormancy release; however, we were unable to clarify the relationship between gene expression levels and these processes. To reveal the molecular mechanisms underlying *P*. *polyphylla* var. *yunnanensis* seed dormancy release during a warm stratification, the transcriptomes of dormant and germinating *P*. *polyphylla* var. *yunnanensis* seeds were separately analyzed by RNA sequencing and were also compared with the transcriptomes of stem-leaf and root tissues harvested during the seed maturation stage. The RNA sequencing of five tissues generated 234,331 unigenes, of which 10,137 (4.33%) were differentially expressed among the analyzed tissues. The 6,619 unigenes whose expression varied among mature dormant, sprouted, and germinated seeds included 95 metabolic and 62 signaling genes related to abscisic acid, gibberellin, auxin, brassinosteroid, cytokinin, ethylene, jasmonic acid and salicylic acid. Additionally, 243 differentially expressed genes were annotated as known seed dormancy/germination-related genes. Among these genes, 109 were regulated by hormones or involved in hormone signal transduction. Finally, 310 transcription factor unigenes, including 71 homologs of known seed dormancy/ germination-related genes, were observed to be differentially expressed during a warm stratification. These results confirm that multiple hormones and transcription factors influence *P*. *polyphylla* var. *yunnanensis* seed dormancy release and germination during a warm stratification. This study identified candidate genes (e.g., *ABI5*) that should be cloned and functionally characterized regarding their effects on the release of *P*. *polyphylla* var. *yunnanensis* seed morphophysiological dormancy.

## Introduction

*Paris polyphylla* var. *yunnanensis* (‘dian chonglou’ in Chinese) is mainly distributed in southwestern China, especially in Yunnan, Sichuan, and Guizhou provinces. This variety is one of two documented *P*. *polyphylla* taxa in the *Pharmacopoeia of the People's Republic of China* (versions 2005, 2010, and 2015). The rhizomes of this plant are the major medicinal organ used alone or in many well-known compound Chinese medicines such as Yunnan Baiyao and Gong Xue Ning. Similar to other *Paris* species, *P*. *polyphylla* var. *yunnanensis* is a slow-growing herbaceous perennial. The fresh weight of rhizomes propagated from mother rhizomes increases by only 9% per year [1]. A previous study revealed that plants propagated from mother rhizome cuttings with terminal buds germinate the following year and grow faster, but rhizome weights are only about 50 g at 3 years after planting [2]. Another study confirmed that plants propagated from seeds grow slowly for up to 4 years after germination, after which they experience a growth spurt [3]. The rhizomes reportedly weigh about 50–100 g at 8 years after germination. Similarly, the contents of four markedly active steroidal saponin compounds (polyphyllin I, II, VI and VII) in the rhizome increase over time, peaking at about 8 years [4, 5]. Because of its long growth period and pressures due to over-collection, the current abundance of wild *P*. *polyphylla* var. *yunnanensis* is inadequate to meet increasing commercial demands. Consequently, *P*. *polyphylla* var. *yunnanensis* has been cultivated during the last few decades.

*Paris polyphylla* var. *yunnanensis* can be propagated from its rhizomes or seeds. Although rhizome propagation can shorten the time required for cultivation, thus giving growers an earlier investment return, it has several shortcomings. One obvious disadvantage is that this approach decreases the quantity of rhizomes available for medicinal use. More importantly, rhizome propagation has a low propagation coefficient. Specifically, a 4–5-year-old rhizome can only provide about three cuttings [6]. Additionally, rhizome cuttings with no terminal buds germinate late and grow slowly [2]. Therefore, seed propagation seems to be a superior alternative to produce seedlings for the large-scale artificial cultivation of *P*. *polyphylla* var. *yunnanensis*. However, *P*. *polyphylla* seeds (*P*. *polyphylla* here and afterwards refers to *P*. *polyphylla* var. *yunnanensis*), which are released from mature capsules, need a long dormancy period (18 months or longer) under natural conditions to complete morphological and physiological processes before germinating. Incompletely developed embryos and inhibitors in the seed coat are thought to be the major factors associated with the morphophysiological dormancy (MPD) of *P*. *polyphylla* var. *yunnanensis* seeds [7–9]. Hormone contents, especially abscisic acid (ABA) and gibberellins (GA) levels, are altered upon the release of seed dormancy release and germination, indicating they help regulate *P*. *polyphylla* var. *yunnanensis* seed dormancy and germination [8]. Temperature is an important environmental factor regulating *P*. *polyphylla* seed MPD. Studies have revealed that seed coat removal, a warm temperature stratification, and a hormone treatment can effectively shorten the seed dormancy period and promote seed germination [6–13]. Mature *P*. *polyphylla* seeds dispersed in the natural habitats of Yunnan province experience two winters (cold conditions) and one summer (warm conditions) before germination. However, a constant exposure to warm conditions (around 20°C) promotes the early germination and relatively rapid radical growth of *P*. *polyphylla* var. *yunnanensis* seeds when compared with cold or alternating cold–warm conditions [7–13].

**Fig 1:**
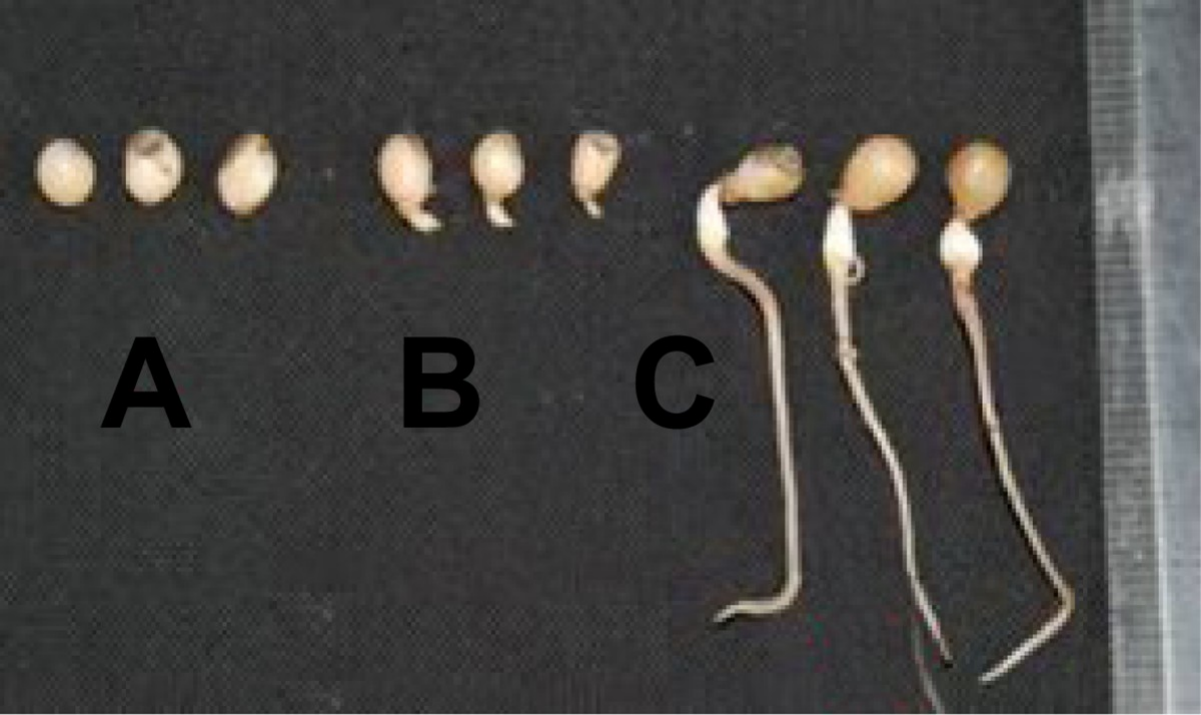
*Paris polyphylla* var. *yunnanensis* seed samples analyzed in this study. (A) Seed: Fresh mature seeds with seed coats removed, dormant before a warm stratification; (B) S_Str: sprouted seeds with 1–2-mm extruded radicles after 6 weeks of a sand stratification; (C) S_Ger: germinated seeds with approximately 5-cm long radicles and no protruded embryos after 14 weeks of a sand stratification.

In an earlier transcriptomic analysis, we proved that genes associated with seed maturation and dormancy release are expressed in *P*. *polyphylla* var. *yunnanensis* seeds following stratification treatments [14]. However, the relationship between the expression of such genes and *P*. *polyphylla* var. *yunnanensis* seed dormancy release remains unknown. To reveal the molecular mechanism underlying *P*. *polyphylla* var. *yunnanensis* seed dormancy release during a warm stratification, we analyzed the transcriptomes of *P*. *polyphylla* var. *yunnanensis* seeds during dormancy and germination stages (Fig 1A–1C) using Illumina RNA sequencing (RNA-seq) technology. As a perennial herb, the rhizomes and the aboveground tissues of *P*. *polyphylla* var. *yunnanensis* must annually undergo seasonal senescence and winter dormancy, which overlaps with its seed maturation and dormancy periods [3]. Similar to seed dormancy, the senescence and dormancy of vegetative tissues in herbaceous perennials is induced/regulated by the combined effects of environmental factors (e.g., temperature) and internal biochemical signals (e.g., hormones) [15]. To clarify whether there is a molecular difference in dormancy initiation among these three tissues, the roots and the aboveground stems and leaves were also collected at the seed maturation stage for subsequent RNA-seq and comparative transcriptome analyses.

## Results and discussion

### *De novo* assembly and functional annotation of the *P*. *polyphylla* transcriptome

*Pari*s *polyphylla* var. *yunnanensis* and var. *chinensis* are two most important *P*. *polyphylla* species in the *Pharmacopoeia of the People's Republic of China*. These two varieties possess the same characteristics of seed dormancy phenomenon, that is, morphological and physiological post-ripening dormancy. Previous observation showed that mature dormant *P*. *polyphylla* var. *chinensis* seeds require approximately 60–80 days to complete embryo development during a warm stratification (18 ± 1°C) [16]. Additionally, 40 days was thought to be an important timepoint for the morphological post-ripening process of dormant *P*. *polyphylla* during a warm stratification. The embryo subsequently grows quickly and the seed metabolic activities are simultaneously enhanced. In the present study, we observed that the radicles in stratified *P*. *polyphylla* var. *yunnanensis* seeds extruded after approximately 6 weeks of a stratification at 20°C, which we assumed to be the onset of seed germination (Fig 1B). After a 14-week stratification, the germinated seeds had about 5-cm long radicles, but no protruded embryos (Fig 1C). We separately collected the stratified germinating seeds at these two timepoints as well as mature dormant seeds to study their gene expression changes during a warm stratification. The transcriptomes of mature seeds (Seed; Fig 1A), stratified seeds (S_Str; Fig 1B), germinated seeds (S_Ger; Fig 1C), a mixture of stems and leaves (StL), and roots (Root) were sequenced with an Illumina Hi-Seq 2500 platform. A total of 298,457,470 clean reads, corresponding to 96.88% of the total raw reads, were generated for these five samples (Table 1). The clean reads were *de novo* assembled with the Trinity program into 311,401 transcripts including 234,331 unigenes. The average transcript and unigene lengths were 610 bp and 541 bp, respectively. A length distribution analysis revealed that more than half of the unigenes/transcripts were shorter than 500 bp (S1 Fig). Of the 196,366 unigenes predicted to encode proteins, 131,892 (56.28%) were identified with ESTScan. The length distributions of the predicted protein-coding unigenes are presented in S2 Fig.

**Table 1:** Overview of *Paris polyphylla* var. *yunnanensis* transcriptome data.

**Total raw reads**	308,053,916
**Total clean raw reads**	298,457,470
**Total clean bases**	37.30Gb
**Q20 percentage**	96.03%
**GC percentage**	49.38%
**Total transcripts**	311,401
**Length range (bp)**	201–16889
**Mean length of transcripts (bp)**	610
**N50(bp)**	912
**N90 (bp)**	253
**Total Unigenes**	234,331
**Total nucleotides (bp)**	126,682,660
**Length rang (bp)**	201–16889
**Mean length of Unigenes (bp)**	541
**N50 (bp)**	738
**N90 (bp)**	238
**No. (%) of total annotated Unigenes**	96,709 (41.27%)
**No. (%) of annotated Unigenes in Nr**	64,313 (27.44%)
**No. (%) of annotated Unigenes in Nt**	44,344 (18.92%)
**No. (%) of annotated Unigenes in Swiss-Prot**	54,119 (23.09%)
**No. (%) of annotated Unigenes in Pfam**	58,558 (24.98%)
**No. (%) of annotated Unigenes in COG**	32,361 (13.80%)
**No. (%) of annotated Unigenes in GO**	59,412 (25.35%)
**No. (%) of annotated Unigenes in KEGG**	27,796 (11.86%)

A total of 96,709 unigenes (41.27%) were annotated based on BLAST searches of the NCBI non-redundant protein (Nr), NCBI non-redundant nucleotide (Nt), Swiss-Prot, Protein family (Pfam), Clusters of Orthologous Group (KOG/COG) of proteins, Gene Ontology (GO), and Kyoto Encyclopedia of Genes and Genomes (KEGG) databases, with 11,870 unigenes annotated with all seven databases (Table 1). A total of 64,313 (27.44%), 44,344 (18.92%), 54,119 (23.09%) and 58,558 (24.98%) unigenes were annotated with the first four databases, respectively. Among the unigenes annotated with the Nr database, 45% displayed 60–80% similarity with their most strongly matched homologs (S3B Fig). The top four matched species were *Phoenix dactylifera* (22.8%), *Musa acuminata* (7.9%), *Vitis vinifera* (4.8%) and *Hordeum vulgare* (3.4%) (S3C Fig), which contrasted with the previous annotations of *P*. *polyphylla* transcriptomes for mature dormant seeds and seed coats [17]. For example, except for *P*. *dactylifera* and *M*. *acuminata*, the top matched species in Ling’s transcriptome data [17] differed from those of our current study. Additionally, our transcriptome include only two homologous unigenes from *Nelumbo nucifera*, which is the third most common species in a previous study [17]. We suspect that these differences in species distributions between two transcriptome annotations of the same plant species may have been due to differences in sequencing depth, assembled unigenes lengths, and especially the studied tissues because different sets of genes are expressed in diverse tissues. Many important developmental processes and traits, including seed dormancy and germination [18–20], have been extensively studied in the important model dicot *Arabidopsis thaliana*. In the current study, 485 unigenes were most similar to *A*. *thaliana* genes in the Nr database.

A BLASTx search of the COG database clustered 32,361 unigenes (13.8%) into 26 categories (S4 Fig). The relative order of the most abundant categories differed slightly from that of one of previous studies [14]. The largest category in the present study was “general functional prediction only” (4,826; 14.91%), which was followed by “translation, ribosomal structure and biogenesis” (4,479; 13.84%) and “post-translational modification, protein turnover and chaperon” (4,191; 12.95%). Moreover, a GO term analysis resulted in 59,412 unigenes being assigned to at least one GO term from one of three main functional categories and 56 subcategories (S5 Fig). The biological process category contained 43,736 unigenes clustered into 23 subcategories, the most abundant of which were “cellular process” and “metabolic process”. Within the cellular component category, 28,534 unigenes were classified into 19 subcategories, the most heavily represented being “cell” and “cell part”. A total of 48,214 unigenes were assigned to 14 molecular function categories, the largest of which was “binding”, followed by “catalytic activity”.

Among 27,796 unigenes (11.86% of 234,331 unigenes) annotated with the KEGG database, 19,990 were mapped by KEGG Mapper (version 2.1) to 318 basic reference pathways in five modules (as of August 2017) (S1 Table). A total of 8,823 unigenes were assigned to the metabolism module, with the six most abundant third-level pathways related to energy, nucleotide, and carbohydrate metabolism, namely oxidative phosphorylation (884 unigenes), glycolysis/gluconeogenesis (664), purine metabolism (589), pyruvate metabolism (523), cysteine and methionine metabolism (476), and amino sugar and nucleotide sugar metabolism (448). The genetic information processing module contained 5,210 unigenes, of which 2,632 genes were involved in the ribosome pathway. Regarding the environmental information processing module, the pathway with the most unigenes was the PI3K-Akt signaling pathway (485). Finally, 297 unigenes were assigned to the plant hormone signal transduction module, which provided us a list of genes for an investigation of the hormone-based regulation of *P*. *polyphylla* seed development and germination.

### Overview of genes differentially expressed among five *P*. *polyphylla* tissues

Gene expression levels of each sample were analyzed by back-mapping their corresponding clean reads to our assembled transcriptomic sequences with RSEM software [21]. Using the default parameters of the Bowtie 2 program, 74.02–77.16% of the clean reads in five samples were mapped to their respective reference sequences. The number of mapped unigenes ranged from 90,345 (Seed) to 138,578 (Root) (S2 Table). The number of fragments per kilobase of transcript sequence per million base pairs sequenced (FPKM), which excludes the effects of sequence length and sequencing depth on gene read counts, was adopted to calculate transcript abundance. Unigenes with FPKM values > 0.3 were considered to be expressed. According to our read remapping results, 15,368 unigenes were not expressed in any tissue, whereas 28,253 were expressed in all five tissues. Of 78,051 unigenes (33.31% of all unigenes) with FPKM values > 0.3, 9,280 were specifically expressed in mature seeds. Among 122,222 unigenes expressed in *P*. *polyphylla* roots, 53,979 were exclusively expressed in roots. The density distribution of FPKM values in the five samples also revealed that the overall gene expression levels of the root sample was distinct from those of three seed samples (Seed, S_Str, and S_Ger) (S6 Fig).

**Fig 2:**
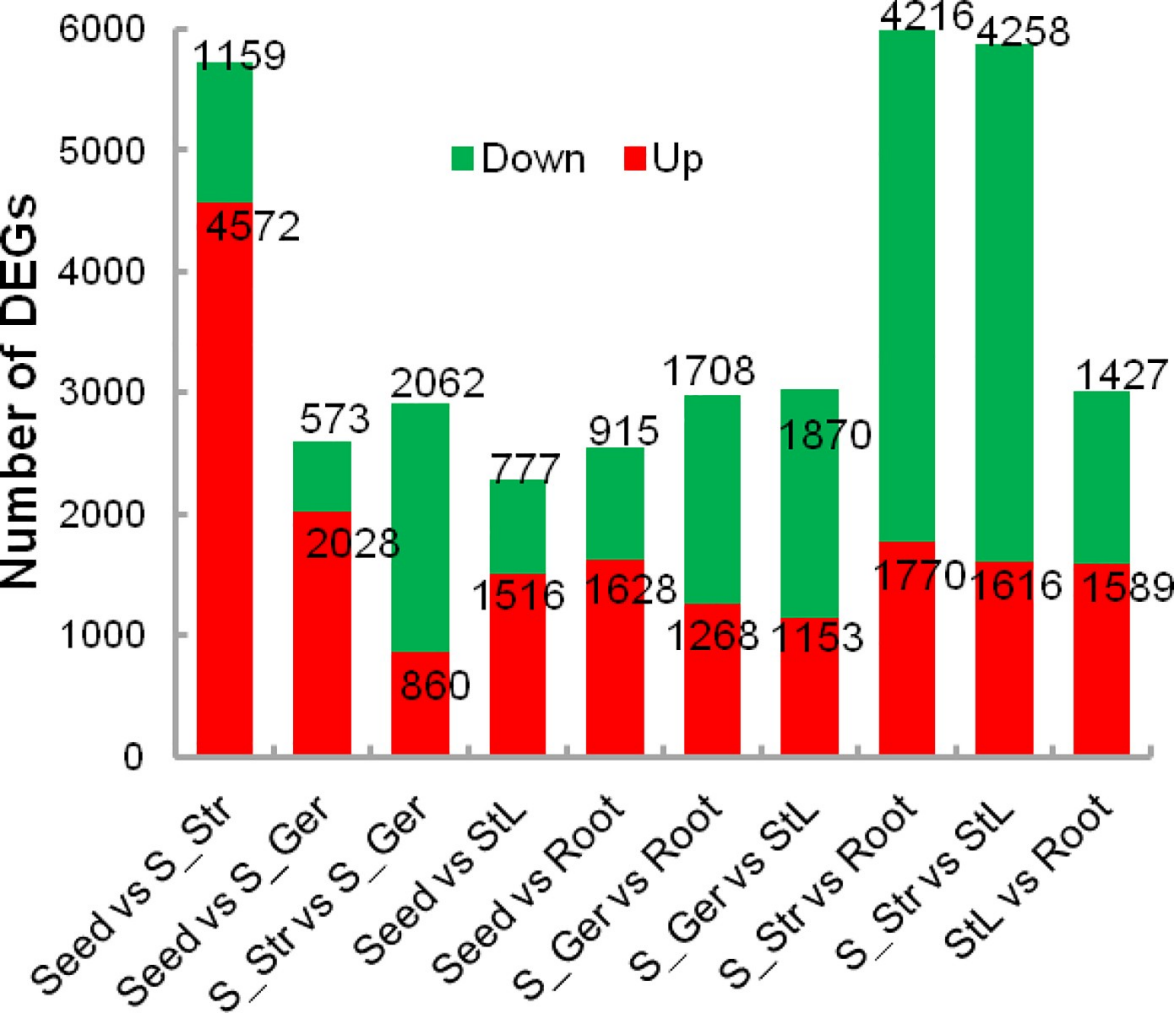
Numbers of differentially expressed genes (DEGs) between pairwise compared tissues. In each pairwise comparison, red and green indicate increased and decreased transcript abundance, respectively, in the first tissue relative to the second.

**Fig 3:**
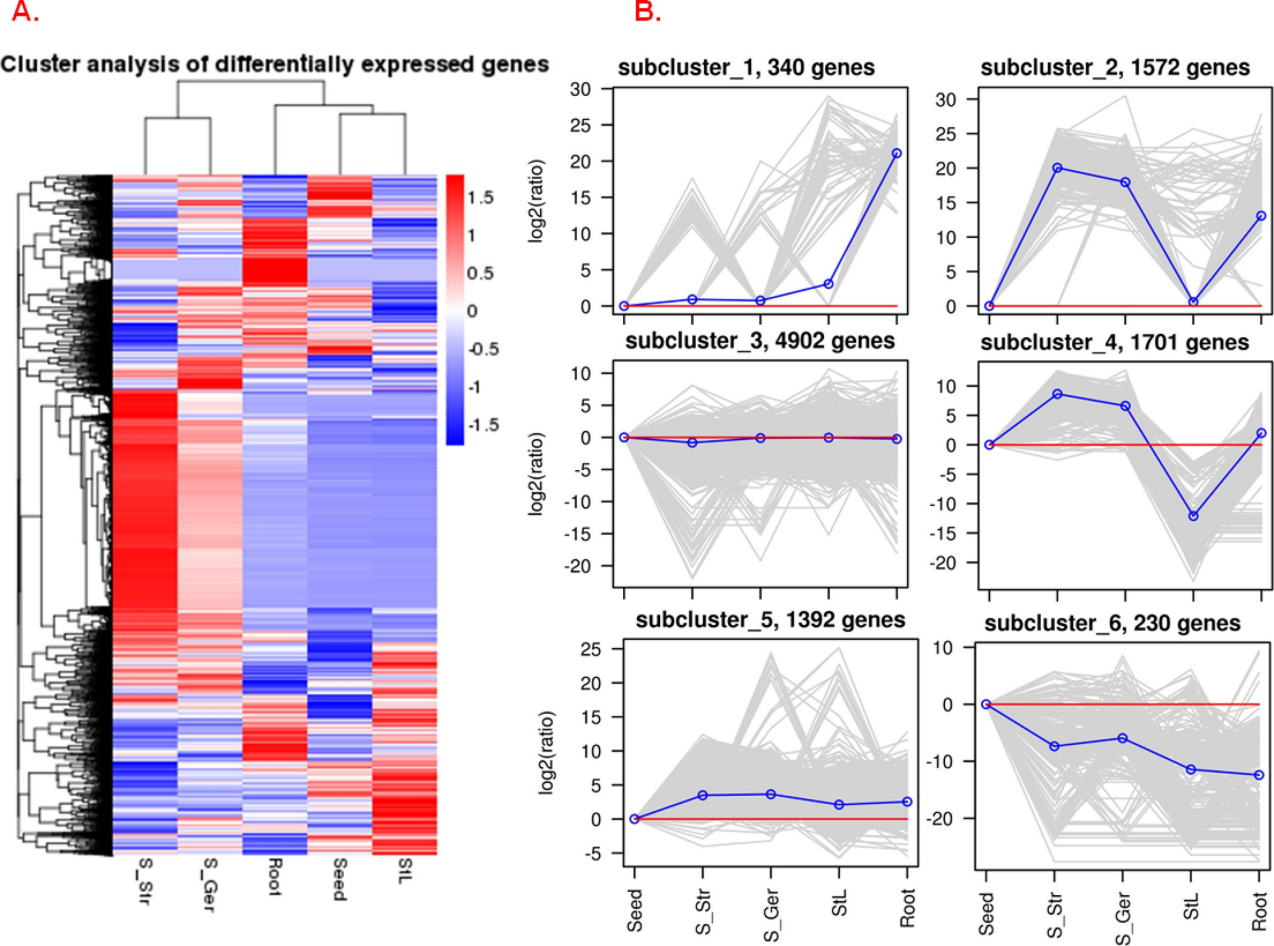
Cluster analysis of 10,137 differentially expressed genes (DEGs) among five *Paris polyphylla* tissues. (A) Hierarchical clustering results. Red and blue represent increased and decreased transcript abundance, respectively. (B) Results of *k*-means clustering. The log_2_ ratio corresponds to the ratio of the log_2_ FPKM values of DEGs in each sample to those in mature seeds.

On the basis of cutoff values of *q* ≤ 0.005 and |log_2_ (fold change)| ≥ 1, a total of 10,137 unigenes were detected as differentially expressed between at least two tissues (S3 Table). The number of differentially expressed genes (DEGs) between each pair of compared tissues is provided in Fig 2. A hierarchical clustering of all DEGs (Fig 3A) grouped S_Str and S_Ger together, and revealed that Root gene expression profiles were closer to those of Seed and StL. These results suggested that the expression of *P*. *polyphylla* genes involved in warm stratification-induced seed germination is quite different from that of dormant seeds and autumnal StL tissue. Furthermore, a *k*-means clustering further classified all DEGs into six sub-clusters based on their log_2_ ratios of four samples to mature seeds (Fig 3B). Sub-cluster 1 contained 340 unigenes that were highly expressed only in *P*. *polyphylla* roots. Sub-clusters 2 and 4 consisted of genes whose transcripts levels increased greatly upon the release of seed dormancy and seed germination, but these genes were not expressed or were expressed at relatively low levels in StL tissue. Sub-cluster 5 included 1,392 unigenes with lower expressions levels in mature seeds than in the other four tissues. The genes in sub-cluster 6 were most highly expressed in mature seeds. Approximately half of the DEGs were grouped into sub-cluster 3, and most of these genes were abundantly expressed at similar levels in all five tissues.

### Differential expression of *P*. *polyphylla* genes in germinating seeds during a warm stratification

**Fig 4:**
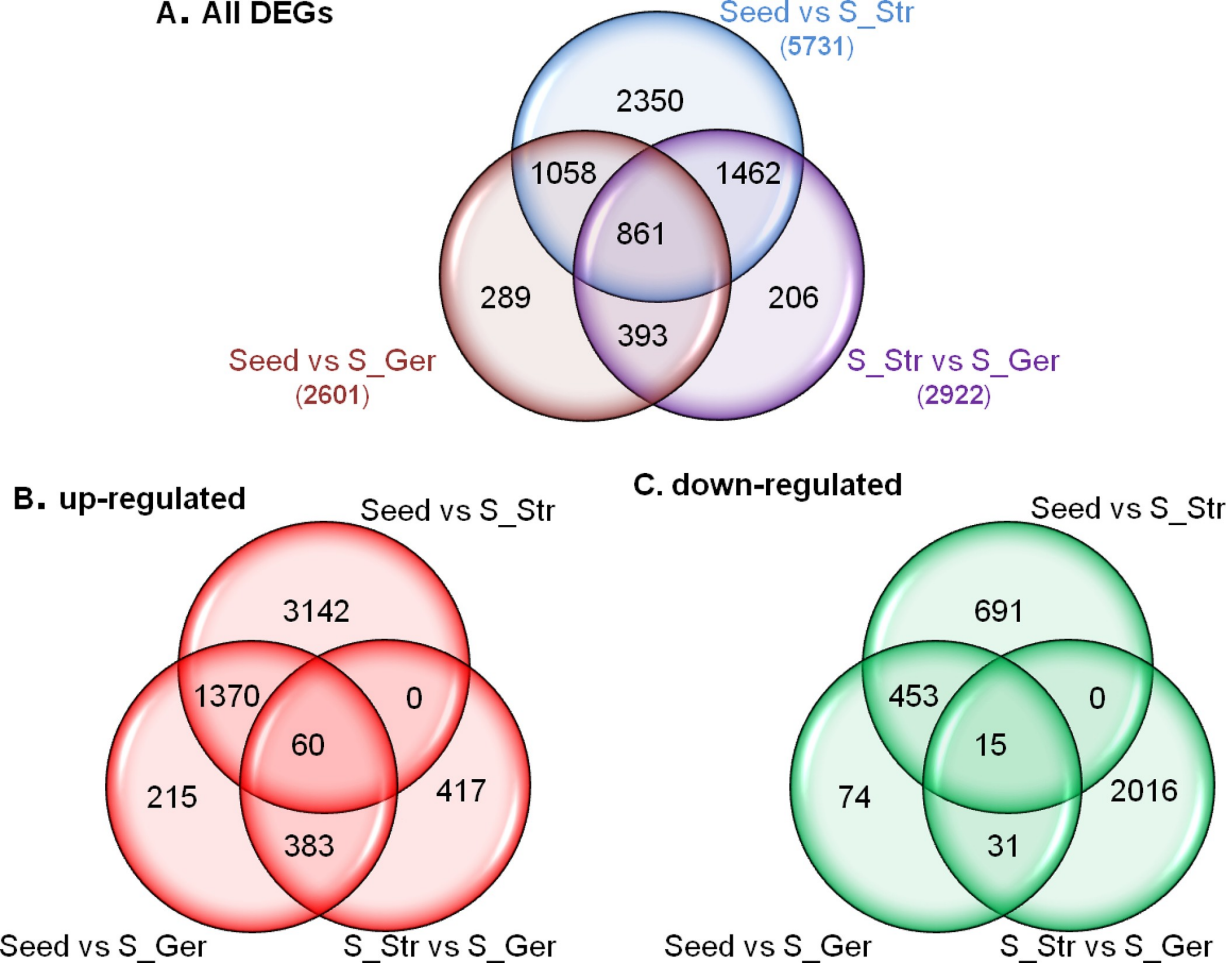
Venn diagrams presenting the numbers of shared or unique differentially expressed genes (DEGs) between dormant and germinating seeds during a warm stratification. In each comparison, the terms up-regulated and down-regulated refer to unigenes in the first tissue exhibiting increased or decreased transcript abundance, respectively, relative to the second tissue.

A total of 6,619 unigenes were differentially expressed among mature dormant seeds (Seed), S_Str and S_Ger (S3 Table, Fig 4A). Of these DEGs, 2,350 (1,875 up-regulated and 475 down-regulated) were detected only between Seed and S_Str, while 206 (179 up-regulated and 27 down-regulated) were uniquely detected between S_Str and S_Ger. Additionally, 289 unigenes (215 up-regulated and 74 down-regulated) were only differentially expressed between Seed and S_Ger. There were 1,058 overlapping DEGs between Seed and S_Str/S_Ger, of which 641 were up-regulated and 417 were down-regulated during seed germination. The comparisons Seed *vs*. S_Str and S_Str *vs*. S_Ger shared 1,462 DEGs, of which 1,260 DEGs were up-regulated in S_Str followed by a significant decrease in S_Ger and 202 DEGs decreased in S_Str and then up-regulated in S_Ger. There were 393 shared DEGs between S_Str *vs*. S_Ger and Seed *vs*. S_Ger. Of these unigenes, 369 were more highly expressed in dormant seeds and S_Str than in S_Ger, while the expression levels of 24 unigenes were down-regulated in germinated seeds after 14 weeks of a warm stratification. Additionally, 861 unigenes were differentially expressed among dormant seeds, S_Str, and S_Ger, and were further classified into six expression pattern categories based on their FPKM values. The major pattern, which was exhibited by 730 unigenes, involved an expression level increase at the onset of germination and then a decrease to levels between those of S_Str and dormant seeds. Although another six unigenes exhibited a similar expression trend, their expression levels in S_Ger were lower than in dormant seeds. Meanwhile, the expression levels of 60 unigenes were continuously up-regulated throughout the seed germination stage, while the expression levels of 15 unigenes were down-regulated during the same stage. The transcript abundances of 14 unigenes decreased in S_Str, but subsequently increased in S_Ger to levels significantly higher than that in dormant seeds. A similar expression trend was observed for 36 other unigenes, their transcript abundances increased considerably after the onset of seed germination, but were still significantly lower in S_Ger than in dormant seeds. Venn diagrams of the distribution of unigenes exhibiting up-regulated or down-regulated expression among three compared seed tissues are provided in Fig 4B and 4C.

The results of our Go annotations and KEGG pathway analysis of 6,619 DEGs are summarized in S3–S9 Tables. The KEGG enrichment results revealed that the plant hormone signal transduction pathway was enriched in S_Str *vs*. S_Ger and Seed *vs*. S_Ger, indicating its importance for seed germination after seed dormancy is released.

#### Differential expression of phytohormone metabolic and signaling genes in germination seeds during a warm stratification

Plant hormones are important for seed dormancy maintenance and release. In previous hormone assays, the ABA contents of stratified *P*. *polyphylla* seeds decreased under a constant or fluctuating warm stratification, whereas the contents of IAA, zeatin riboside (ZR), and especially GA increased markedly with the release of seed dormancy and the initiation of germination [22–24]. A BLASTx search of an *A*. *thaliana* protein database revealed 663 metabolic genes in our transcriptomic database that are involved in biosynthetic/degradation pathways of ABA, GA, auxin, brassinosteroid (BR), cytokinin (CK), ethylene, jasmonic acid (JA), salicylic acid and strigolactone (S10 Table). Moreover, 137 unigenes involved in the biosynthesis/degradation of nine hormones were differentially expressed among the five studied tissues, of which, 95 were differentially expressed in seed samples during a warm stratification (S11 Table). Furthermore, a KEGG analysis annotated 297 *P*. *polyphylla* unigenes as plant hormone signaling genes, of which 294 had *A*. *thaliana* homologs (S12 Table). A BLASTx search of hormone signaling genes described in the published literature and *A*. *thaliana* homologs ultimately uncovered 458 putative *P*. *polyphylla* hormone signaling unigenes (S12 Table). Of these putative hormone signaling genes, 103 exhibited tissue-specific differential expression, while 62 were differentially expressed among three seed samples during a warm stratification (S13 Table). The expressions levels of metabolic and signaling genes related to ABA, GA, auxin, BR, CK, ethylene, JA, and salicylic acid are described in more detail below.

**Fig 5:**
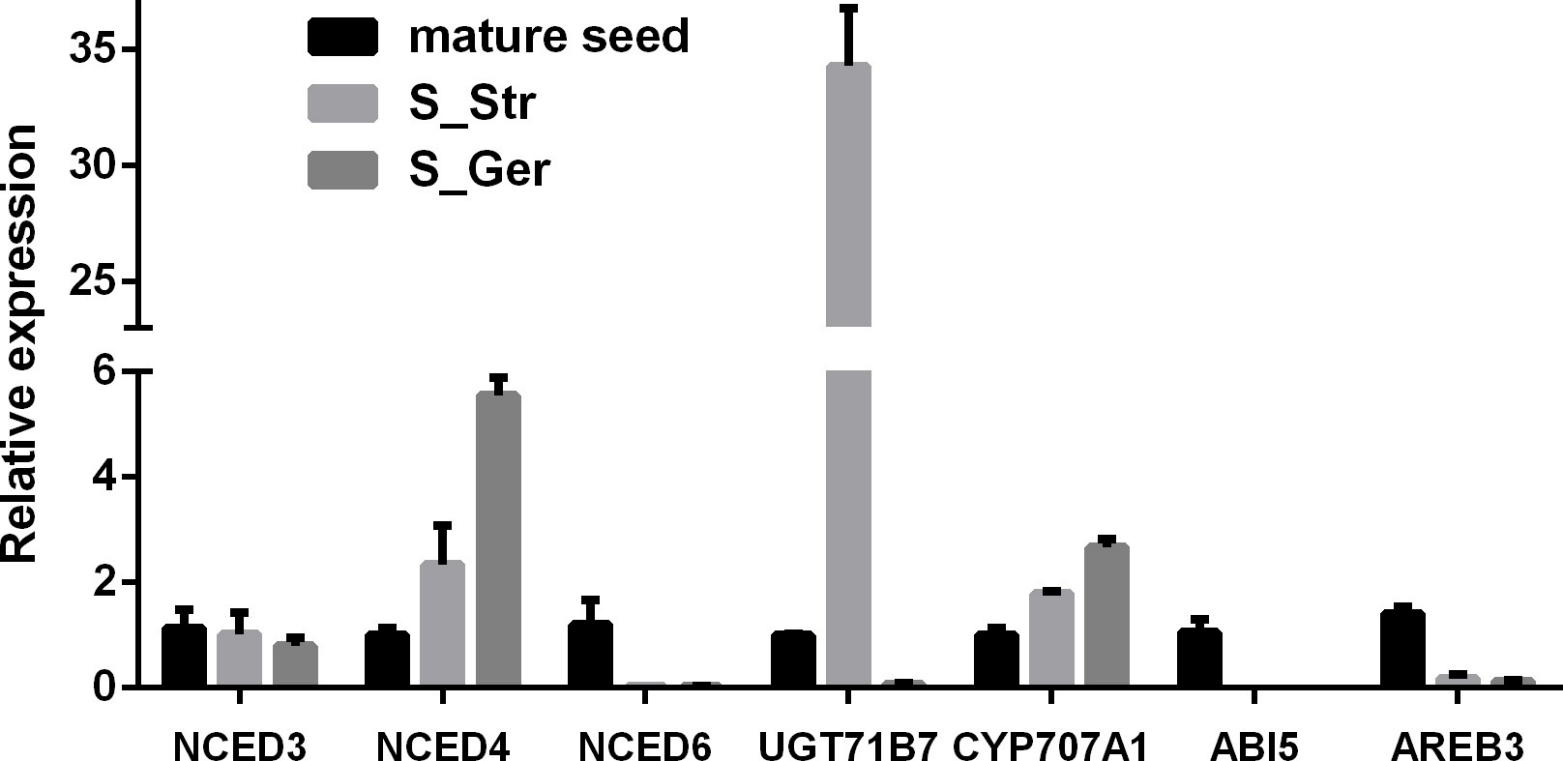
Validation of selected ABA metabolic and signaling genes by RT-qPCR. Three *NCED* genes, two ABA degradation genes and *ABI5* and *AREB3* were validated by RT-qPCR to confirm their expressions during a seed stratification. The genes on the x-axis were named based on their *Arabidopsis thaliana* homologs (S14 Table).

**Abscisic acid metabolic and signaling genes:** The endogenous ABA concentration is determined by the balance between ABA biosynthesis and catabolism. We identified 47 ABA biosynthetic unigenes and 18 ABA catabolic unigenes in our dormant or senescent tissue transcriptomes (S10 Table), indicating the simultaneous occurrence of ABA biosynthesis and catabolism in these tissues. Six unigenes involved in ABA biosynthesis were differentially expressed during the warm stratification of seed, namely one down-regulated *NPQ1* homolog and five xanthoxin dehydrogenase genes (three up-regulated and two down-regulated) (Table 2). The ABA content of stratified *P*. *polyphylla* seeds decreased with seed dormancy release during a warm stratification [22–24], implying the catabolism of ABA overwhelmed its biosynthesis. Abscisic acid degradation involves conjugation and hydroxylation pathways. For example, ABA uridine diphosphate glucosyltransferase (UGTase) converts active ABA to an inactive ABA-glucose form, and thus plays a critical role in the modulation of cellular ABA levels. The *A*. *thaliana* genome contains three highly similar ABA *UGT* genes (*UGT71B6*, *UGT71B7*, and *UGT71B8*), the expressions of which lowers ABA levels and is negatively correlated with seed germination rates [25]. One candidate ABA UGTase unigene (c128611_g1; *AtUGT71B7* homolog) was identified in our *P*. *polyphylla* transcriptome database, and its expression was markedly induced during seed dormancy release and germination (Table 2, Fig 5). We also observed that the transcripts levels of two ABA hydroxylation genes (c139767_g1 and c148601_g2) were greater in stratified seeds than in dormant seeds. These results indicated that the decrease in ABA content of stratified *P*. *polyphylla* seeds was mainly due to the significantly up-regulated expression of ABA catabolic genes, thus indicating their possible roles in breaking the *P*. *polyphylla* seed MPD and inducing germination in response to a warm stratification.

**Table 2:** Abscisic acid metabolic and signaling genes that were differentially expressed during a warm stratification.

Unigene ID	At homolog name	log2ratio (Seed vs S_Str)	log2ratio (S_Str vs S_Ger)	log2ratio (Seed vs S_Ger)
**Biosynthetic**				
c138815_g1	AT2G21860.1	-7.2641		-7.1404
c139345_g1	ABA2;;SDR1	2.5423		1.6507
c140928_g2	ABA2;;SDR1		3.6112	
c144287_g1	ABA2;;SDR1	-3.0318		
c155036_g1	AT3G26760.1	8.9238	-1.7596	
c141040_g1	SDR2	3.3072		3.0086
**Catalytic**				
c128611_g1	UGT71B7	5.1995	-1.4065	3.6447
c139767_g1	CYP707A1	4.4691	1.0739	5.3947
c148601_g2	CYP707A4	3.3597	-3.679	
**signaling gene**				
c134152_g1	PYR1; RCAR11	2.6689		2.4193
c126034_g1	PYL4; RCAR10	7.3818		6.3168
c138003_g1	PYL8; RCAR3	1.2196		
c138003_g2	PYL9; RCAR1	2.5632		1.688
c142214_g1	HAB1	-1.0348		
c143278_g1	HAI1; SAG113	-4.5797		-3.657
c146828_g1	AHG3; PP2CA	1.621		2.24
c149313_g1	AHG3;PP2CA	-4.339		-3.7871
c141271_g1	SNRK2.5; MBK5.13; SRK2H	1.4604		
c151879_g1	AREB3	-2.1513	-1.4677	-3.7673
c144664_g1	AREB3			-2.7176
c140327_g2	AREB3			-2.0926
c144660_g1	ABI5	-5.7511		-6.6842

More information, including BLASTx results and FPKM values, is available in S10–S13 Tables.

The core ABA signaling pathway consists of the PYR/PYL/RCAR family of ABA receptors, a negative regulator of ABA signaling protein type 2C phosphatases (PP2Cs), a positive regulator of its downstream genes, the SUCROSE NONFERMENTING1-RELATED SUBFAMILY2 (SnRK2) protein, and ABA-responsive genes. Of 19 differentially expressed ABA signaling genes, 13 had markedly altered transcript abundances in stratified seeds relative to morphophysiologically dormant seeds, namely six up-regulated unigenes (four *PYR/PYL/RCAR*s, one *PP2C*, and one *SnRK2*) and seven down-regulated unigenes (three *PP2Cs* and four *ABFs*) (Table 2, S13 Table). The four *P*. *polyphylla PYR/PYL/RCAR* unigenes were homologous to *A*. *thaliana PYR1*, *PYL4*, *PYL8*, or *PYL9*, the first three of which are down-regulated by ABA [26, 27]. The four *P*. *polyphylla PP2C*s unigenes were homologous to *A*. *thaliana HAB1*, *HAI1*, and *AHG3*, which are all up-regulated by ABA [26, 28]. The *A*. *thaliana* genome contains 14 *PYR/PYL/RCAR* genes and nine Clade-A PP2C genes. These genes are associated with multiple mechanisms that modulate ABA signaling and response via differential gene expression in specific tissues or developmental stages, different combinations of ABA receptor complexes with different affinities, or alteration to the ratio of receptor to PP2C [26, 28]. Our results did not immediately reveal how (and which of) these PYR/PYL/RCARs and PP2Cs in stratified seeds coordinately regulate the ABA signaling pathway via changes to their expression levels. However, our data helped identify candidate genes for further investigations of the molecular mechanisms underlying the occurrence and release of *P*. *polyphylla* seed MPD. Two *AHG3* homologs (c146828_g1 and c149313_g1) were differentially expressed during a warm stratification, suggesting their functions differ during various tissue developmental stages. More specifically, the up-regulated expression of c146828_g1 in stratified seeds is indicative of its positive effects on seed sprouting and germination during a warm stratification, whereas the down-regulated expression of c149313_g1 implies this gene is associated with the establishment of *P*. *polyphylla* seed MPD. Future investigations of the expression dynamics of these two *AHG* homologs during *P*. *polyphylla* seed development and maturation may further characterize their contribution to *P*. *polyphylla* seed MPD. *Arabidopsis thaliana* ABI5 is a key bZIP transcription factor (TF) that helps regulate seed maturation, dormancy, and germination via the linking of the ABA signaling pathway with other signaling molecules, including GA/auxin/BR/CK, NO and radicle oxidative stress (ROS) [29–31]. Although two *P*. *polyphylla ABI5* unigenes were identified in search of an *A*. *thaliana* protein database for homologs, only c144660_g1 was highly expressed in dormant seeds (FPKM = 1769.34 based on RNA-seq), while it was expressed at considerably lower levels in the other four tissues (FPKM = 3.73–95.43) (S13 Table). The RT-qPCR results further confirmed its high expression profile in mature dormant seeds (Fig 5).

**Fig 6:**
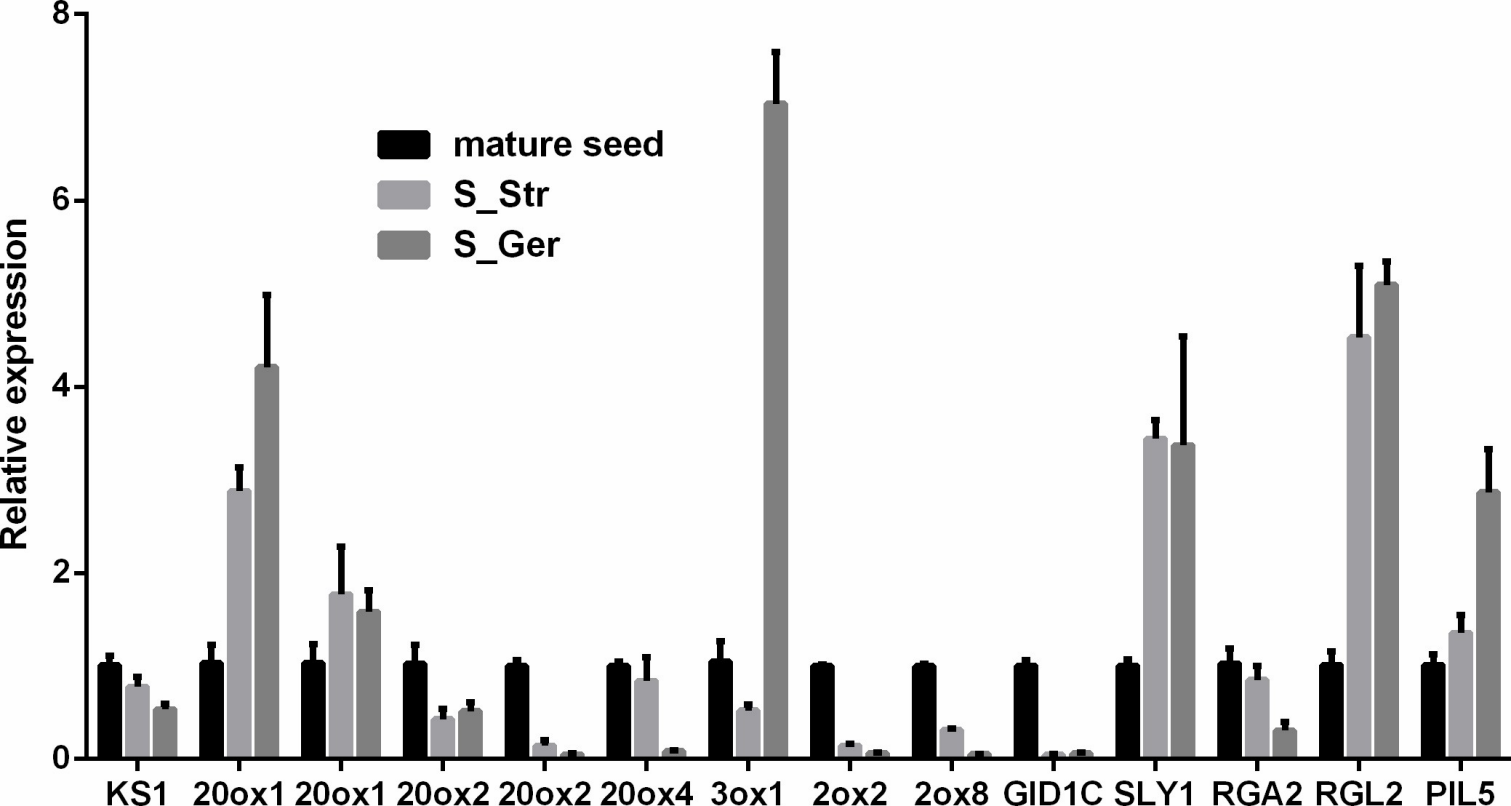
Results of an RT-qPCR analysis of selected GA metabolic and signaling genes. Fourteen genes (eight DEGs and six GA synthetic genes that were not differentially expressed) were analyzed. The genes on the x-axis were named based on their *A*. *thaliana* homologs (S14 Table).

**Gibberellin metabolic and signaling genes:** Previous studies revealed that exogenous GA shortens the *P*. *polyphylla* seed dormancy period and promotes seed germination in wet sand during a temperature stratification [32, 33]. Additionally, the endogenous GA content reportedly increases in stratified seeds [22–24]. In the current study, FPKM-based transcription levels indicated that GA metabolic genes were expressed at relatively low levels in dormant seeds and stratification-induced sprouted and germinated seeds (S11 Table). However, six GA metabolic unigenes were differentially expressed between stratified and dormant seeds (Table 3). The expression level of one unigene encoding *ent*-kaurenoate oxidase (c112970_g1) in the GA biosynthetic pathway was up-regulated in sprouted and germinated seeds, whereas the expression levels of two other GA biosynthetic unigenes encoding *ent*-kaurene synthase (c152785_g1) and gibberellin 20-oxidase (c137729_g1) were down-regulated. In the GA catabolic pathway, the expression of two GA2ox unigenes was up-regulated during a warm stratification. An earlier investigation proved that *A*. *thaliana GAMT2*, which encodes a GA9 carboxyl methyltransferase that converts active GA to inactive forms, is mainly expressed in developing seeds, with relatively low expression levels in mature and germinating seeds [34]. We identified one *P*. *polyphylla GAMT2* unigene, and observed that its expression was down-regulated in stratified seeds. This down-regulation may be related to increased GA levels in stratified seeds, and therefore, may promote the final development of *P*. *polyphylla* seed embryos and dormancy release. In the GA signaling pathway, the *GID1* GA receptor family genes and the F-box protein gene *SLY1* encode positive regulators of seed germination, whereas DELLA proteins are negative regulators [35, 36]. The expression levels of one *GID1C* homolog and one *SLY1* unigene were up-regulated in stratified seeds (Table 3). The expression of one unigene annotated as a homolog of the DELLA protein gene *GAI* was down-regulated in S_Str, but was subsequently up-regulated in S_Ger during a stratification. Meanwhile, the transcript abundance of c141357_g1, which was annotated as the *A*. *thaliana* DELLA protein gene *RGL2*, increased throughout the stratification. Moreover, *A*. *thaliana PIL5* negatively regulates light-dependent seed germination through ABA and GA signaling [37]. The expression of one *PIL5* unigene was up-regulated up until the late stratification period, implying it influences root and embryo growth after the release of seed dormancy. Additionally, 14 genes (eight DEGs and six GA synthetic genes that were not differentially expressed) revealed by RNA-seq were further analyzed by RT-qPCR (Fig 6).

**Table 3:** Gibberellin metabolic and signaling genes that were differentially expressed during a warm stratification.

Unigene ID	At homolog name	log2ratio (Seed vs S_Str)	log2ratio (S_Str vs S_Ger)	log2ratio (Seed vs S_Ger)
**Biosynthetic**				
c152785_g1	KS1	-3.2385		-2.9562
c112970_g1	KAO1; CYP88A3	5.936	-2.3677	
c137729_g1	GA20ox2	-4.7778		
**Catalytic**				
c123261_g1	GA2ox2		1.4149	
c134079_g1	GA2ox8	3.6225	-1.5532	1.9209
c143307_g1	GAMT2	-5.4165		-2.8869
**Signaling gene**				
c144250_g1	GID1C	1.8657	-1.2398	
c152084_g1	SLY1	1.5827		
c143401_g1	GAI;RGA2	-2.6536	2.4337	
c141357_g1	RGL2	1.2902		1.0953
c152411_g1	PIF1; PIL5		2.0077	

More information, including BLASTx and FPKM values, is available in S10–S13 Tables.

**Auxin metabolic and signaling genes:** A BLASTx search of an *A*. *thaliana* protein database identified 121 auxin anabolic unigenes, 25 auxin degradation-catalyzing unigenes, and 250 auxin signaling unigenes (S10 and S12 Tables). During a seed stratification, the expression levels of three auxin biosynthetic unigenes (*IAR3*, *YUC10* and *CYP71A22*) were down-regulated, whereas the expression levels of 12 auxin biosynthetic unigenes and four auxin catalytic unigenes were up-regulated (Table 4). A previous study involving the application of exogenous hormones revealed that *P*. *polyphylla* seed germination is promoted by specific exogenous IAA concentrations [32]. Additionally, the endogenous IAA content in stratified *P*. *polyphylla* seeds decrease at the beginning of a stratification and subsequently increases until the seeds sprout [24]. We believe that the simultaneous up-regulated expresion of auxin anabolic genes and IAA/IBA-conjugating genes observed in this study was correlated with this fluctuation, which may regulate auxin homeostasis to maintain auxin at a suitable level for different physiological conditions of seeds during a stratification. Of 43 auxin signaling genes differentially expressed among the five studied *P*. *polyphylla* tissues, the expression levels of 15 [one auxin efflux gene, two auxin influx genes (*AUX1* and *LAX3*), two *IAA* family genes, six *ARF* genes, three *GH3*s and one *SAUR* gene] were higher in S_Str and/or S_Ger than in dormant seeds (S13 Table, Table 4). The expressions levels of six *ARF* unigenes homologous to the *A*. *thaliana* adventitious-rooting positive regulators *ARF6*, *ARF8* [38], and *ARF8* decreased in sprouted seeds, but then increased in germinated seeds. A homolog of *ARF17*, which encodes a negative regulator of *A*. *thaliana* adventitious rooting [38], and three unigenes encoding negative regulators of auxin signaling (two *IAA16* homologs and o ne *IAA31* homolog) exhibited down-regulated expression during a warm stratification. Overall, our results provide evidence that *P*. *polyphylla* seed dormancy release and germination during a warm stratification is tightly correlated with the down-regulated expression of genes encoding negative regulators of auxin signaling and the up-regulation expression of auxin biosynthetic genes, auxin transporters genes, and genes encoding positive regulators of auxin signaling.

**Table 4:** Auxin metabolic and signaling genes that were differentially expressed during a warm stratification.

Unigene ID	At homolog name	log2ratio (Seed vs S_Str)	log2ratio (S_Str vs S_Ger)	log2ratio (Seed vs S_Ger)
**Biosynthetic**				
c141974_g1	iar3			-1.0837
c192256_g1	iar3	5.4034		
c10273_g1	YUC4	7.5978	-3.5588	
c156668_g1	YUC4	9.3764	-2.6046	6.6235
c104057_g1	YUC10	7.2766	-2.4018	
c147047_g1	YUC10	-2.4695	2.74	
c135580_g1	CYP71A23			4.2997
c190547_g1	NIT2	12.401	-2.0317	10.221
c140182_g1	NIT4		1.1331	
c78877_g1	CYP71A15	4.7231		
c111863_g1	CYP71A20	5.0984		
c141339_g1	CYP71A22	-9.7257		-8.5316
c152621_g1	CYP71A25	1.1796		
c143632_g1	CYP71A26	2.7411		1.6964
c148707_g2	CYP71A26		2.4619	5.5836
**Catalytic**				
c142848_g1	GH3.1		2.3774	2.8743
c153937_g1	GH3.1	2.3838	-2.4551	
c142848_g2	GH3.3		2.5926	2.8421
c148016_g1	UGT74E2	3.052	-1.0822	1.8215
**Signaling gene**				
c151672_g1	PIN7			2.9008
c143837_g1	AUX1; MAP1; PIR1; WAV5	2.7471		
c137523_g1	LAX3			5.5372
c153883_g3	ARF1			1.0794
c140235_g2	ARF1			2.0138
c139686_g2	ARF2;ARF1-BP;HSS; ORE14	1.2165		
c151595_g1	MP;ARF5;IAA24	2.7991		1.8964
c105833_g1	ARF6	-4.3736	2.5162	-2.0058
c135065_g1	ARF6	-1.9023		
c151001_g1	ARF6	-1.4952	1.4797	
c112366_g1	ARF8	-4.3641		
c138760_g3	ARF8	-4.2151		
c148686_g2	ARF9		1.5301	1.7781
c122217_g1	ARF17	-1.0418		-2.1802
c144687_g1	ARF18		2.5665	
c153567_g2	ARF19;IAA22			1.0507
c132474_g1	IAA16	-5.2118		-3.9871
c137001_g1	IAA16	-3.303		-1.9835
c147875_g1	IAA26;PAP1		2.7399	3.6555
c133318_g1	IAA27;PAP2		1.8736	1.349
c142426_g1	IAA31	-5.8257		-6.2166
c142848_g1	GH3.1		2.3774	2.8743
c153937_g1	GH3.1	2.3838	-2.4551	
c142848_g2	GH3.3		2.5926	2.8421
c141114_g2	SAUR31	3.2205		3.6414

More information, including BLASTx results and FPKM values, is available in S10–S13 Tables.

**Brassinosteroid metabolic and signaling genes:** *Arabidopsis thaliana* genes encoding enzymes involved in campesterol-derived BR biosynthesis include *DET2*, *CYP724*, *CYP85*, and *CYP90* family members [39]. Of the 36 *P*. *polyphylla* unigenes annotated as BR biosynthetic genes (S10 Table), the expression of one *CYP85A1* unigene was up-regulated during a stratification, whereas the expression levels of two *CYP90B1* unigenes were first down-regulated in sprouted seeds and then up-regulated in germinated seeds (S11 Table). Among 24 BR-inactivating unigenes, the transcript abundances of three unigenes (*CYP709B2*, *UGT73D1* or *BAS1*) were markedly increased in sprouted seeds and then decreased to various degrees in germinated seeds. The expression of *CYP85A1*, which encodes a protein that catalyzes the later steps of brassinolide biosynthesis, is feedback-regulated and is reportedly up-regulated in a *CYP90B1*-containing triple mutant [40]. In the RNA-interference triple mutant, a decreas in *CYP90B1*-*DET2*-*SMT2* transcription was observed to alter the contents of BR intermediates, but not seed germination. Another study revealed that the over-expression or up-regulated expression of tomato *CYP85A1* accelerates seed germination [41]. Additionally, *BAS1* encodes a brassinolide C-26 hydroxylase that decreases active BR levels and modulates seed germination independently and/or downstream of PHYA and PHYB [42]. However, no BR signaling genes were differentially expressed during the *P*. *polyphylla* seed stratification in the current study (S12 and S13 Tables).

**Cytokinin metabolic and signaling genes:** The endogenous ZR content of *P*. *polyphylla* seeds increases markedly during a seed stratification [24]. Our homology analysis uncovered 38, 47, and 7 *P*. *polyphylla* unigenes possibly involved in CK biosynthesis, conjugation and inactivation, respectively (S10 Table). The expression of one of four *IPT5* unigenes was induced in sprouted seeds, but was then subsequently down-regulated in germinated seeds (Table 5). The expression of one *LOG1* homolog was significantly enhanced in germinating seeds. Additionally, CYP715A1 was predicted by AarCyc to catalyze the formation of the *trans*-zeatin intermediates *trans*-zeatin riboside di/triphosphates. On the base of research involving *A*. *thaliana cyp715a1* mutant, CYP715A1 modulates GA and JA homeostasis [43]. In our study, a unigene homologous to *CYP715A1* (56.34% sequence identity) was more highly expressed in sprouted and germinating seeds than in mature seeds. Although the ZR content increases in stratified *P*. *polyphylla* seeds [24], we observed that the expression levels of 12 predicted CK-conjugating genes and two CK-degrading genes (*CKX2/3)* were up-regulated during a stratification. Only one CK-conjugating gene (c150218_g1; *UGT73C5* homolog) and one *CKX1* homolog exhibited down-regulated expression in sprouted and/or germinating seeds. Some CKX-encoding genes as well as two CK-conjugating genes (ZOG genes) are reportedly more highly expressed in post-ripened seeds than in dormant seeds during an imbibition [44]. The rice zeatin *O*-glucosyltransferase gene (*OscZOG1*) is preferentially expressed in shoot and root meristematic tissues and nascent organs [45]. Additionally, the overexpression of *AtCKX2* promotes early seed germination [46]. These findings indicate that the increased transcription of CK-conjugating and CK-degrading genes together with CK biosynthetic genes possibly contributes to *P*. *polyphylla* seed dormancy release and germination during a warm stratification. In *A*. *thaliana*, CK signaling involves the positive regulators CK receptor histidine kinases (AHKs), histidine phosphotransfer proteins (AHPs), and response regulators [47]. Forty-three *P*. *polyphylla* unigenes were annotated as putative CK signaling genes (S12 Table). The expression levels of two CYTOKININ-INDEPENDENT unigenes (*CKI1* and *CKI5*) and one *AHK3* gene were up-regulated in sprouted seeds, whereas the expression levels of five other CK signaling unigenes (*AHK4*, *AHP1*, *ARR9*, *CYCLIN D3*, and *CYCLIN D2*) were down-regulated in sprouted seeds, but up-regulated in germinating seeds (Table 5). A study of *A*. *thaliana* mutants proved that the expression of *CRE1*/*AHK4* together with *AHK2* and *AHK3* greatly suppresses seed germination in darkness, thereby indicating these genes negatively regulate seed germination [46].

**Table 5:** Cytokinin metabolic and signaling genes that were differentially expressed during a warm stratification.

Unigene ID	At homolog name	log2ratio (Seed vs S_Str)	log2ratio (S_Str vs S_Ger)	log2ratio (Seed vs S_Ger)
**Biosynthetic**				
c140303_g1	IPT5	5.9846	-6.0831	
c128988_g1	LOG1		3.3543	1.4469
c153068_g1	CYP715A1	4.5151		4.4657
**Inactivation/degradation**				
c139436_g1	AT2G36770.1	2.5607		
c136084_g1	AT2G36780.1		1.9146	1.9701
c146109_g1	AT2G36780.1	6.6074		
c151142_g1	AT2G36780.1	4.0813	-2.5118	1.4212
c153261_g1	UGT85A2	3.716		3.6066
c150519_g1	UGT85A1	2.3321		2.0199
c143984_g2	UGT73C1	3.1763		
c138207_g2	DOGT1;UGT73C5	5.4569	-1.8615	3.4471
c143984_g4	DOGT1;UGT73C5	2.4289		
c143984_g5	DOGT1;UGT73C5	3.8133	-2.5208	
c147689_g1	DOGT1;UGT73C5			1.2521
c150218_g1	DOGT1;UGT73C5		1.7691	
c152465_g1	DOGT1;UGT73C5	3.685	-2.1723	1.3645
c147606_g1	CKX1	-4.765		-3.2875
c100492_g1	CKX2	11.083	-2.5257	8.4085
c83626_g1	CKX3	7.533	-1.8165	5.6762
**Signaling gene**				
c86190_g1	CKI1	4.8166		
c1038_g1	AHK5;CKI2	5.2776		
c18449_g1	AHK3	5.4222		
c153218_g1	CRE1;AHK4		1.2799	
c141184_g1	AHP1	-2.4539		
c112256_g1	AHP1	-2.2565		
c139287_g2	ARR9	-3.0566		
c148489_g1	CYCLIN D3;2	-3.0723	3.2168	

More information, including BLASTx results and FPKM values, is available in S10–S13 Tables.

**Ethylene metabolic and signaling genes:** Ethylene is synthesized from methionine following the sequential activities of the following three enzymes: methionine adenosyltransferase (MAT or SAMS), 1-aminocyclopropane-1-carboxylase synthase (ACS) and 1-aminocyclopropane-1-acid carboxylic oxidase (ACO) [48]. Additionally, 1-aminocyclopropane-1-carboxylic acid (ACC) is the direct biosynthetic precursor of ethylene, and promotes the germination of dormant *P*. *polyphylla* seeds [49]. Of 86 unigenes annotated as ethylene biosynthetic genes, the expression levels of two *MAT* genes, one *ACS1* gene, and three *ACO* genes were up-regulated during a seed stratification, while the expressions levels of two *ACO5* unigenes were down-regulated in sprouted seeds and then slightly up-regulated afterwards (S10 and S11 Tables). Ethylene signaling in *A*. *thaliana* involves ethylene receptor genes [ethylene response 1 (*ETR1*), *ETR2*, ethylene response sensor 1 (*ERS1*), *ERS2*, and ethylene insensitive 4 (*EIN4*)], the negative regulator *CTR1* (constitutive triple response), and the positive regulator *EIN2* as well as its downstream nuclear TFs such as *EIN3* (ethylene insensitive), *EIL*s (EIN3-like), *ERBP*s (ethylene responsive element-binding protein), and *ERF*s (ethylene response factor) [48]. In our study, 32 unigenes were identified as homologs of *A*. *thaliana* ethylene signaling genes (S12 Table). The expression levels of only two unigenes corresponding to *EBF1* and *ERF1* were up-regulated in germinating seeds during a seed stratification (S13 Table).

**Jasmonate metabolic and signaling genes:** Jasmonates promote or inhibit seed germination depending on the specific jasmonate or plant type [50, 51]. A previous study confirmed that JA biosynthesis begins in the chloroplast with the conversion of linolenate to 12-oxo-phytodienoate (OPDA) by the sequential activities of lipoxygenase (LOX), allene oxide synthase (AOS) and allene oxide cyclase (AOC) [50]. The generated OPDA is then transported by the ATP binding cassette (ABC) transporter COMATOSE (CTS) into the peroxisome and finally degraded into JA and its derivatives by the sequential activities of OPDA reductase (OPR), carboxyl-CoA ligase (OPCL1), acyl-CoA oxidases (ACX), multifunctional proteins (AIM1 and MFP), L-3-ketoacyl-CoA thiolase (KAT), and JAR1. A BLASTx search identified 114 *P*. *polyphylla* unigenes homologous to these *A*. *thaliana* JA biosynthetic genes (S10 Table). The expression levels of 18 and 3 of these genes were respectively up-regulated and down-regulated in sprouted and/or germinating seeds during a warm stratification (S11 Table). Although exogenous MeJA decreases the germination rates of dormant *P*. *polyphylla* seeds [49], the two unigenes homologous to *A*. *thaliana JMT* identified in the present study were not differentially expressed during a warm stratification. Up-regulated expression levels of JA biosynthetic genes have also been observed in coleorhiza of imbibing barley seeds [52] and in wheat seeds imbibed after ripening [53]. Among 18 JA signaling unigenes uncovered in our study (S12 Table), the expression levels of four of five DEGs (*JAR1*, *JAZ*, and *JAI*) were up-regulated during a warm stratification (S13 Table), consistent with the increased expression of JA biosynthetic unigenes. The collective up-regulated expression of JA biosynthetic and signaling unigenes implies that jasmonates participate in *P*. *polyphylla* seed dormancy release; however, elucidating their exact roles and the mechanism regulating *P*. *polyphylla* seed dormancy release will require further research, including the profiling of individual jasmonate metabolites as well as analysis of synergistic or antagonistic relationships with other hormones.

**Salicylic acid metabolic and signaling genes:** The phenylalanine ammonia lyase (PAL)-mediated phenylalanine pathway and the isochorismate synthase (ICS)-mediated isochorismate pathway are the two known SA biosynthesis pathways. A BLASTx search identified 1 *ICS*, 24 *PAL*, and 11 SA-glucosylation unigenes (S10 Table). Of these genes, the expression levels of three *PAL* and one *UGT74F2* unigenes were up-regulated during a warm stratification (S11 Table). Among the 36 SA signaling unigenes (S12 Table), the transcriptions of three (NPR1-like *BOP2*, *TGA9*, and PR1-like) were enhanced during a warm stratification (S13 Table). The *TGA2* and *TGA6* genes are essential for triggering the SA-mediated suppression of JA/ET-induced defense responses [54] and to impede *COI1*-mediated root growth inhibition imposed by phytoprostanes [55]. The expression levels of two unigenes corresponding to these two closely related TGA TFs were down-regulated during a warm stratification.

#### Expression of functionally characterized seed dormancy/ germination-related genes

**Fig 7:**
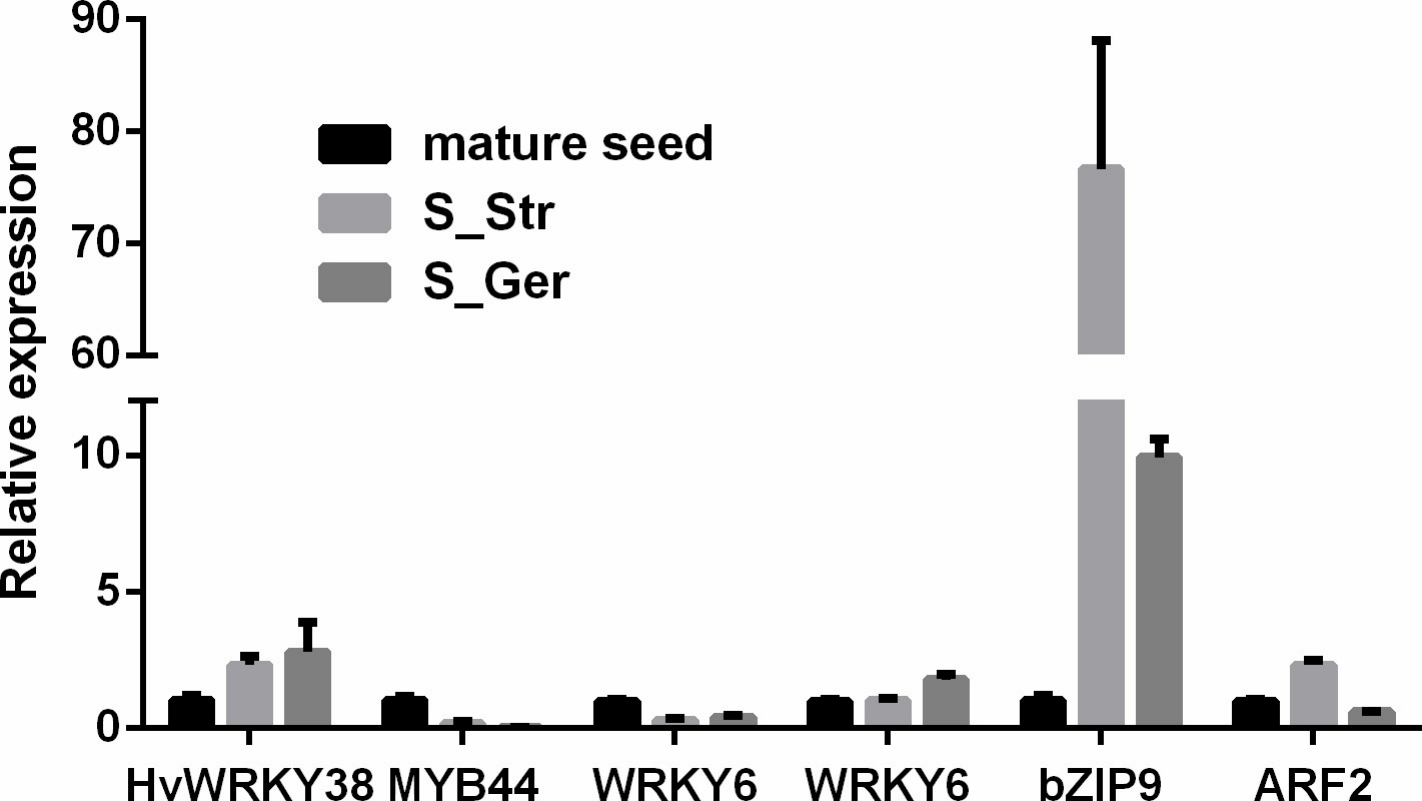
Validation of selected functionally characterized seed dormancy/germination-related genes by RT-qPCR. The genes on the x-axis were named based on their *Arabidopsis thaliana* homologs (S14 Table).

Although comparisons of dormant and germinating seeds in *A*. *thaliana*, barley, rice, maize, and other crops have revealed thousands of DEGs possibly regulating seed dormancy and germination, our literature search only uncovered 530 genes functionally characterized as having regulatory roles in these processes (S15 Table). A BLAST search identified 326 differentially expressed unigenes as homologs of 219 known seed dormancy/germination-related genes (S16 and S17 Tables). Of these 326 DEGs, 186 were differentially expressed among seeds, stems and leaves, and roots harvested during the seed maturation stage (S17 Table, column B). The expression levels of 243 of these unigenes were regulated during a warm stratification, including 157 enhanced/decreased 86 unigenes in sprouted and/or germinated seeds relative to dormant seeds (S18 Table). Of these 243 DEGs, 30 TF unigenes were differentially expressed between germinating seeds and dormant or senescent samples (i.e., mature seeds, stems and leaves, and roots). These observations imply these genes are important for seed germination during a warm stratification. Six TF genes were validated by RT-qPCR (Fig 7). A total of 109 DEGs were regulated by hormones or involved in hormone signaling pathways. The expression patterns of most unigenes were consistent with the characterized functions of their corresponding known homologs in seed dormancy/germination. For example, *AtPER1* encodes an antioxidant related to seed dormancy, and its homolog in *P*. *polyphylla*, unigene c144307_g1, was abundantly expressed in mature dormant seeds (FPKM = 1310.6) before exhibiting considerably down-regulated expression during a warm stratification (FPKM = 22.42–1.13). Moreover, *AtS2*, *TaSDR1B*, *TaPM19-A2*, *OsqLTG3-*1, and *GmMFT* are also considered to be seed dormancy genes, and the expressions of their corresponding *P*. *polyphylla* homologs were down-regulated during a warm stratification for seed dormancy release. Soluble sugar, soluble protein, and starch contents reportedly decrease in *P*. *polyphylla* seeds during the stratification process [56]. In the current study, we determined that the expression levels of storage matter-related metabolism genes changed accordingly. For example, the expression levels of c150256_g1 (homolog of the galactinol synthase gene *LeGOLS-1*), c145361_g1 (homolog of the starch-debranching enzyme *OsPUL*), c153006_g1 (homolog of the protein mobilization gene *PsTPE4A*) and c128491_g1 (homolog of the lipid-transfer protein gene *ElLTP2*) were induced or up-regulated to help mobilize seed storage matter in germinating seeds.

### Differential expression of *P*. *polyphylla* genes among three tissues during the seed maturation stage

**Fig 8:**
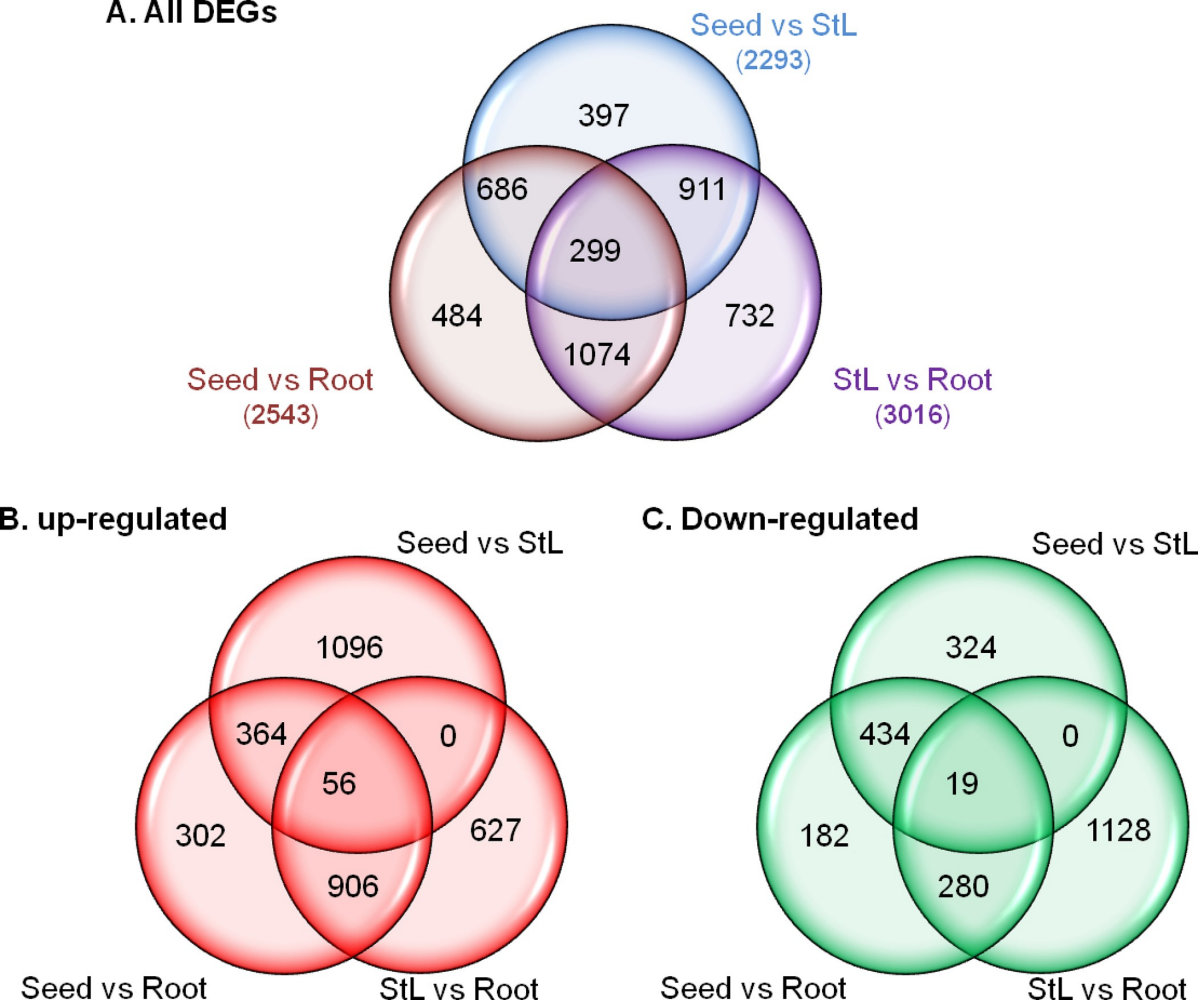
Venn diagrams presenting numbers of shared or unique DEGs among three *Paris polyphylla* tissues collected during the seed maturation stage. In each comparison, the terms up-regulated and down-regulated refer to unigenes whose expression levels in the first tissue are respectively higher and lower than the corresponding expression levels in the second tissue.

The aboveground tissues of *P*. *polyphylla* become senescent around October, which is when seeds mature, and wither in November. Rhizomes, as perennial vegetatively propagating tissues, also reach their growth peak rates at this time and initiate seasonal winter dormancy. To reveal the molecular differences in the dormancy initiation of these three tissues, we compared their transcriptome during the seed maturation stage. Of 4,583 unigenes differentially expressed among Seed, StL, and Root samples, the expression levels of only 299 unigenes underwent significant changes in all three analyzed tissues (Fig 8A). A total of 484, 732 and 397 DEGs were specific to Seed *vs*. Root, StL *vs*. Root, and Seed *vs*. StL, respectively. Additionally, 686 unigenes were differentially expressed between the seed and both Root and StL, while 1,074 were differentially regulated between the Root and both Seed and StL samples. The expression levels of 911 unigenes in the StL sample were significantly different from those of both Seed and Root. Separate Venn diagram analyses of unigenes exhibiting up-regulated or down-regulated expression revealed that the expressions levels of 56 unigenes, from highest to lowest, followed the order of Root > StL > Seed, whereas the expression levels of 19 Unigenes, from lowest to highest, were in the order of Root < StL < Seed (Fig 8B and 8C). The GO annotation and KEGG analysis of the DEGs among these three tissues was summarized in S4–S7 Tables. A KEGG enrichment analysis of all DEGs revealed that 28, 24, and 20 dorminant pathways were respectively detected in Seed *vs*. StL, Seed *vs*. Root, and StL *vs*. Root (S7 Table). The following 10 enriched pathways were shared across the three pairs of tissue comparisons: “metabolic pathways”, “biosynthesis of secondary metabolites”, “photosynthesis”, “photosynthesis-antenna proteins”, “sphingolipid metabolism”, “ubiquinone and other terpenoid-quinone biosynthesis”, “zeatin biosynthesis”, “phenylpropanoid biosynthesis”, “plant hormone signal transduction” and “plant-pathogen interaction”. A total of 83 unigenes involved in the metabolism of ABA, GA, auxin, CK, BR, ethylene, JA, and SA were differentially expressed among the mature seeds, stems and leaves, and roots (S19 Table), including 56 unigenes whose expression was affected by stratification-induced germination. Finally, 69 unigenes involved in ABA, GA, auxin, CK, BR, ethylene, JA, and SA signaling pathways were differentially expressed among mature seeds, stems and leaves, and roots (S20 Table), including 37 unigenes with altered expressions during stratification-induced germination. These data imply that the hormonal regulation of *P*. *polyphylla* seed maturation/dormancy differs from that of *P*. *polyphylla* StL and root senescence during the seed maturation stage.

### Identification of *P*. *polyphylla* transcription factors and analysis of their differential expression among five tissues

Transcription factors play important roles in the regulation of the seed dormancy/ germination and seasonal senescence/dormancy of perennial plant tissues [57–59]. A BLAST search of an *A*. *thaliana* protein database identified 2,678 *P*. *polyphylla* unigenes homologous to *A*. *thaliana* TF genes (S21 Table). Additionally, 507 of these unigenes belonging to 67 TF families were differentially expressed among the analyzed tissues, and were classified by *k*-means cluster analysis into six clusters containing 1, 41, 300, 32, 110, and 23 DEGs (Fig 2B, Table 6 and S22 Table). Moreover, 311 unigenes were differently regulated among the three tissues harvested during the seed maturation stage. The expression levels of 310 unigenes belonging to 58 TF families were modulated in seeds during a stratification, with the expression of 90 and 220 unigenes respectively lower higher in sprouted and/or germinating seeds than in mature dormant seeds. Furthermore, 38 modulated unigenes belonging to nine TF families were involved in the signal transduction of plant hormones such as ABA and auxin (S22 Table). Finally, 71 TF DEGs belonging to 23 TF families were homologs of functionally characterized genes involved in regulating seed dormancy/germination (S18 Table).

**Table 6:** Number of differentially expressed *Paris polyphylla* transcription factor genes in different *k*-means clusters.

Cluster No.*	DEGs	TF family (No. of DEGs)
1	1	MADS(1)
2	41	Orphans(6), C2H2(4), PHD(3), SNF2(3), CCAAT(3), MYB-related(3), MYB(2), Jumonji(2), SET(2), HSF(1), SWI/SNF-BAF60b(1), C3H(1), G2-like(1), AP2-EREBP(1), HMG(1), FAR1(1), HB(1), GNAT(1), BSD(1), SOH1(1), CSD(1), C2C2-GATA(1)
3	300	HB(25), C3H(18), ARF(15), bHLH(13), NAC(12), bZIP(12), MYB(12), GRAS(12), AP2-EREBP(12), Orphans(10), MYB-related(9), C2H2(8), GNAT(7), PHD(7), C2C2-CO-like(6), WRKY(6), Jumonji(6), C2C2-Dof(5), ABI3VP1(5), SET(5), AUX/IAA(5), FHA(5), Trihelix(5), zf-HD(4), CCAAT(4), HSF(4), BBR/BPC(4), mTERF(4), FAR1(4), Tify(3), TCP(3), C2C2-GATA(3), HMG(3), CSD(3), MADS(3), E2F-DP(3), G2-like(3), Sigma70-like(3), GRF(2), LIM(2), TRAF(2), Tub(2), RB(2), PLATZ(2), ARID(2), RWP-RK(2), ARR-B(2), PBF-2-like(1), SWI/SNF-BAF60b(1), Alfin-like(1), SNF2(1), LOB(1), LUG(1), Coactivator p15(1), MED6(1), EIL(1), SBP(1), CAMTA(1)
4	32	C2H2(8), Orphans(4), C3H(3), PHD(3), SNF2(3), HB(2), HSF(2), SET(2), AP2-EREBP(1), HMG(1), IWS1(1), SWI/SNF-SWI3(1), TRAF(1)
5	110	WRKY(19), NAC(13), AP2-EREBP(13), C2H2(8), TRAF(7), MYB(6), HB(3), CCAAT(3), bHLH(3), bZIP(3), GNAT(3), TCP(2), G2-like(2), LOB(2), Tify(2), Orphans(2), HSF(2), ULT(1), C2C2-YABBY(1), MADS(1), ABI3VP1(1), Tub(1), MBF1(1), DBP(1), Sigma70-like(1), C2C2-GATA(1), AUX/IAA(1), C3H(1), Jumonji(1), GRAS(1), Pseudo ARR-B(1), ARF(1), GRF(1), CSD(1)
6	23	bZIP(5), MADS(3), CCAAT(3), AUX/IAA(2), C2C2-Dof(2), MYB(2), ABI3VP1(1), C3H(1), C2H2(1), SNF2(1), bHLH(1), HMG(1)

*Cluster number based on the *k*-means clusters in Fig 2B.

## Conclusions

A warm stratification greatly shortens the duration of *P*. *polyphylla* seed dormancy and also marginally enhances the seed germination rate. In this study, a comparative transcriptomic analysis of multiple tissues revealed the altered expression levels of many genes during a warm stratification. Plant hormones, especially ABA, GA, and auxin, are important for the maintenance and release of seed dormancy. Transcription factors also have major regulatory functions related to seed dormancy/germination and seasonal senescence/dormancy of perennial plant tissues. Our BLASTx search of an *A*. *thaliana* protein database resulted in the identification of 663 metabolic genes involved in ABA, GA, auxin, BR, CK, ethylene, JA, salicylic acid, and strigolactone biosynthetic/degradation pathways. Of 137 unigenes differentially expressed among five tissues, 95 associated with the biosynthesis/degradation of nine hormones were differentially expressed in seed samples during a warm stratification. Additionally, 103 of 458 putative *P*. *polyphylla* hormone signaling unigenes exhibited tissue-specific differential expression, of which 62 were differentially expressed among three seed samples during a warm stratification. The changes in the expression of most of the 243 DEGs annotated as known seed dormancy/germination-related genes were consistent with the known functions related to seed dormancy maintenance /release. Consequently, these genes are candidates for future studies. Finally, 310 TF unigenes, including 71 homologs of known seed dormancy/germination-related genes, were also differentially expressed during a warm stratification. Our results indicate the involvement of multiple hormones and TFs in the regulation of *P*. *polyphylla* seed dormancy release and germination during a warm stratification.

## Materials and methods

### Plant materials, RNA extraction, cDNA library construction, and sequencing

*Paris polyphylla* var. *yunnanensis* plants were grown in Wuding, Yunnan province, China. Fresh tissues, including mature seeds, roots, and a mixture of stems and leaves, were separately collected in October 2015 and frozen in liquid nitrogen for a subsequent RNA extraction. To break the seed dormancy and promote embryo development, a second batch of fresh mature seeds harvested at the same time was stored in wet sand in a temperature-controlled incubator at 20°C as a warm stratification treatment. Seeds were retrieved for an RNA isolation after a stratification of approximately 6 weeks and 3 months and were respectively named as S_Str and S_Ger samples.

Total RNA was extracted from the collected samples with an RNeasy Plant kit (BioTeke, Beijing, China) and treated with DNase I (Promega, WI, USA) to remove contaminating DNA. The isolated RNA was quantified using a Qubit RNA Assay kit and a Qubit 2.0 fluorometer (Life Technologies, CA, USA), and its integrity and purity were checked on a 1% agarose gel. The RNA was extracted from 60 seeds for the Seed, S_Str, and S_Ger samples as well as from roots (Root) and stems and leaves (StL) of 10 plants. Each RNA sample was shipped to Beijing Novogene Bioinformatics Technology Co. (Beijing, China) for the construction of Illumina mRNA libraries and subsequent sequencing. The libraries were generated with the NEBNext Ultra RNA Library Prep Kit for Illumina (NEB, USA). Briefly, mRNA was purified from 3 μg of DNase I-treated total RNA using oligo(dT) magnetic beads and then separated into short fragments with the divalent cations in NEBNext fragmentation buffer. The cleaved mRNA fragments were reverse transcribed into first-strand cDNA with random hexamer primers, which was followed by second-strand cDNA synthesis using DNA polymerase I and RNase H. The resulting double-stranded cDNA samples were purified with an AMPure XP system (Beckman Coulter, Beverly, USA) and then subjected to blunt-end repair as well as the addition of a poly(A)-tail and the ligation of a sequencing adapter. The ligated products were separated by agarose gel electrophoresis, and suitable fragments were size-selected using the AMPure XP system and then used as templates for a PCR amplification. The resulting PCR libraries were purified, quantified by ABI real-time RT-PCR, and qualified on an Agilent 2100 Bioanalyzer. The libraries were then sequenced on an Illumina HiSeq2500 platform (Illumina, San Diego, CA, USA) to generate 100-bp paired-end reads. The raw reads generated in this study have been deposited in the SRA database (SRR5947180, SRR5947181, SRR5947182, SRR5947183, and SRR5947184).

### Reference transcriptome *de novo* assembly and annotation

Clean reads for the five sequenced tissues were used for the *de novo* assembly of the *P*. *polyphylla* reference transcriptome. Clean reads were obtained by removing raw reads containing adapters, reads with more than 10% unknown nucleotides, and low-quality reads with more than 50% of the bases with a Phred quality scores ≤ 20. Reads shorter than 50 bp or with 70% of their bases having a quality scores ≥ 20 were also excluded from the clean reads. The default parameters of the Trinity program (http://trinityrnaseq.github.io) were applied for the sequence assembly [21]. The assembled transcripts were used as the reference sequences for the *P*. *polyphylla* transcriptome. The longest transcript of each gene was regarded as the unigene for subsequent analyses. The assembled unigenes were submitted to NCBI TSA database (GHCK00000000).

Unigenes in the *P*. *polyphylla* reference transcriptome were annotated by BLASTn or BLASTx searches of the following seven databases: Nr, Nt, Pfam, Swiss-Prot, KOG/COG, GO, and KEGG. The BLAST searches of Nr, Nt, KOG/COG, and Swiss-Prot databases were completed with the NCBI BLAST package (2.2.30+) with a hit threshold of *e* ≤ 1×10^−5^. Meanwhile, the BLAST searches of the Pfam, GO, and KEGG databases were performed using the HMMER 3.0 (*e* ≤ 0.01), Blast2GO v25 and in-house scripts (*e* ≤ 1×10^−6^), and the KAAS program (*e* ≤ 1×10^−10^), respectively. The coding sequence region and direction of each unigene were determined according to the matches in the Nr and Swiss-Prot databases. When a unigene could not be aligned to sequences in any of the abovementioned databases, the default parameters of ESTScan (v3.03) were used to predict the coding region and direction.

### Analysis of gene expression levels and differential expression

The FPKM values that were used to estimate gene transcript abundances in individual samples were obtained with the default parameters of the RSEM program (version1.2.15) [60]. If a gene had multiple transcripts, its expression level was calculated based only on its longest transcript (unigene).

To analyze the differential expression between two tissues, the read counts of the unigenes in the sequenced library were adjusted according to the TMM normalization method in the edgeR package (version 3.0.8), after which the DEGs were identified with the DEGSeq package (version1.12.0) [61]. Genes with an adjusted *p*-value ≤ 0.005 and |log_2_ (fold change)| ≥ 1 were considered to be significantly differentially expressed between two compared tissues.

The GO enrichment analysis of DEGs was completed with the GOseq R package (version1.10.0). The GO terms with corrected *p*-values ≤ 0.05 were considered to be significantly enriched. Additionally, the AmiGO program (http://amigo1.geneontology.org/) was used to determine the lowest level of an enriched GO term. The KEGG pathway enrichment analysis of DEGs was completed with a hypergeometric distribution test followed by a Benjamini-Hochberg correction. The enriched KEGG pathways were detected based on a corrected *p*-value cutoff of ≤ 0.05.

### Identification of hormone metabolism genes, transcription factor genes, and seed germination-related genes in the *P*. *polyphylla* transcriptome

Candidate *P*. *polyphylla* hormone metabolism genes and TF genes were identified by a BLASTx search of an *A*. *thaliana* protein database (Araport11_genes.201606.pep.repr.fasta), which was completed with a local blast program(blast-2.2.30+). The best hit was used to identify *A*. *thaliana* homologs of *P*. *polyphylla* genes. Lists of *A*. *thaliana* hormone metabolism genes and TF genes were respectively obtained from http://www.plantcyc.org/databases/aracyc/15.0 and http://plntfdb.bio.uni-potsdam.de/v3.0/. Plant genes with known functions in seed germination or dormancy were obtained from studies included in the NCBI PubMed database (S15 Table). The encoded protein sequences were used in BLAST searches to identify *P*. *polyphylla* homologous genes possibly contributing to the release of seed dormancy and seed germination during a warm stratification.

### Validation of representative genes by RT-qPCR

Total RNA was separately extracted from three replicates of Seed, S_str, and S_Ger samples with the RNA Extraction kit (Aidlab, China). The purified RNA was used as the template for a reverse transcription, which was completed in a 50-μL reaction volume containing 4 μg of DNase I-treated total RNA, transcriptase (Takara, China) and oligo-(dT)_18_ primer. The RT-qPCR was completed with the ABI 7500 system (Applied Biosystems, USA), with a 10-μL reaction volume consisting of 5 μL 2× SYBR Premix Ex *Taq*, 0.2 μL 50× ROX dye, 0.5 μL 4-fold diluted cDNA, 0.2 μL each primer (10 μM), and 3.9 μL distilled deionized water. Amplification conditions were as follows: 95°C for 30 s; 40 cycles of 95°C for 5 s and 60°C for 34 s. Because ABA and GA play major roles in seed dormancy and germination, 21 genes (of which 13 differentially expressed) related to ABA/GA metabolism and signaling pathways were validated by RT-qPCR. Another six DEGs annotated as homologs of known seed-dormancy/germination genes were also validated by RT-qPCR. The housekeeping gene Actin7 was used as the reference gene [49]. Genes and the gene-specific primer sequences are provided in S14 Table.

## Supporting information

S1 Fig Length distribution of the transcripts and unigenes assembled from Illumina paired-end reads for *P. polyphylla* transcriptomes. (TIF)

S2 Fig Length distribution of protein-coding unigenes predicted based on a BLAST search and the ESTScan program. (TIF)

S3 Fig Homology analysis of *P. polyphylla* unigenes annotated based on the Nr database. (TIF)

S4 Fig Classification of *P. polyphylla* unigenes based on the COG database. (PDF)

S5 Fig Classification of *P. polyphylla* unigenes based on GO terms. (TIF)

S6 Fig Distribution of FPKM values for five *Paris polyphylla* tissues. The x-axis and y-axis represent the gene log_10_ (FRKM) values and densities, respectively. Seed, S_Str, S_Ger, StL, and Root (here and afterward) correspond to mature seed (Fig 1A), stratified seed (Fig 1B), germinating seed (Fig 1C), stem and leaf, and root samples, respectively.
(TIF)

S1 Table Pathway annotation of *P. polyphylla* unigenes in the transcriptome. (XLSX)

S2 Table Summary of the sequencing data of five *P. polyphylla* tissues and the associated gene expression levels. (XLSX)

S3 Table Details regarding the 10,137 differentially expressed genes among five *P. polyphylla* tissues. (XLSX)

S4 Table Enriched GO terms of differentially expressed unigenes in *P. polyphalla* tissues (Q-value ≤0.05). (XLSX)

S5 Table Enriched GO terms of unigenes exhibiting up-regulated expression in *P. polyphalla* tissues (Q-value ≤0.05). (XLSX)

S6 Table Enriched GO terms of unigenes exhibiting down-regulated expression in *P. polyphalla* tissues (Q-value ≤0.05). (XLSX)

S7 Table Enriched KEGG pathways of differentially expressed unigenes among *P. polyphalla* tissues (Q-value ≤0.05). (XLSX)

S8 Table Enriched KEGG pathways of unigenes exhibiting up-regulated expression among *P. polyphalla* tissues (Q-value ≤0.05). (XLSX)

S9 Table Enriched KEGG pathways of unigenes exhibiting down-regulated expression among *P. polyphalla* tissues (Q-value ≤0.05). (XLSX)

S10 Table List of *P polyphylla* hormone metabolism genes identified by a BLASTx search of an *Arabidopsis thaliana* protein database. (XLSX)

S11 Table Differential expression of *P. polyphylla* unigenes involved in hormone metabolism in different tissues. (XLSX)

S12 Table List of *P. polyphylla* hormone signaling genes identified by a BLASTx search of Arabidopsis thaliana protein database. (XLSX)

S13 Table Differential expression of plant hormone signaling genes among five *P. polyphylla* tissues. (XLSX)

S14 Table *Paris polyphylla* genes validated by RT-qPCR. (XLSX)

S15 Table Known genes involved in plant seed dormancy and germination. (XLSX)

S16 Table BLASTx search results for *P. polyphylla* DEGs homologous to known plant genes involved in seed germination and dormancy. (XLSX)

S17 Table Differential expression of putative *P. polyphylla* unigenes homologous to known plant seed germination/dormancy-related genes in different tissues. (XLSX)

S18 Table Differential expression of putative *P. polyphylla* seed germination/dormancy-related unigenes during a warm stratification. (XLSX)

S19 Table Differentially expressed *P. polyphylla* hormone metabolic unigenes among three tissues harvested during the seed maturation stage. (XLSX)

S20 Table Differential expression of plant hormone signaling genes among three *P. polyphylla* tissues harvested during the seed maturation stage. (XLSX)

S21 Table *Paris polyphylla* putative transcription factors. (XLSX)

S22 Table Differential expression of *P. polyphylla* putative transcription factors genes in different tissues. (XLSX)
