# Supplementary figures and images for: Comparative transcriptomic analysis reveals genes regulating the germination of morphophysiologically dormant Paris polyphylla seeds during a warm stratification
Source: PLoS One. 2019 Feb 21;14(2):e0212514. doi: 10.1371/journal.pone.0212514 (PMC6383930; doi:10.1371/journal.pone.0212514)

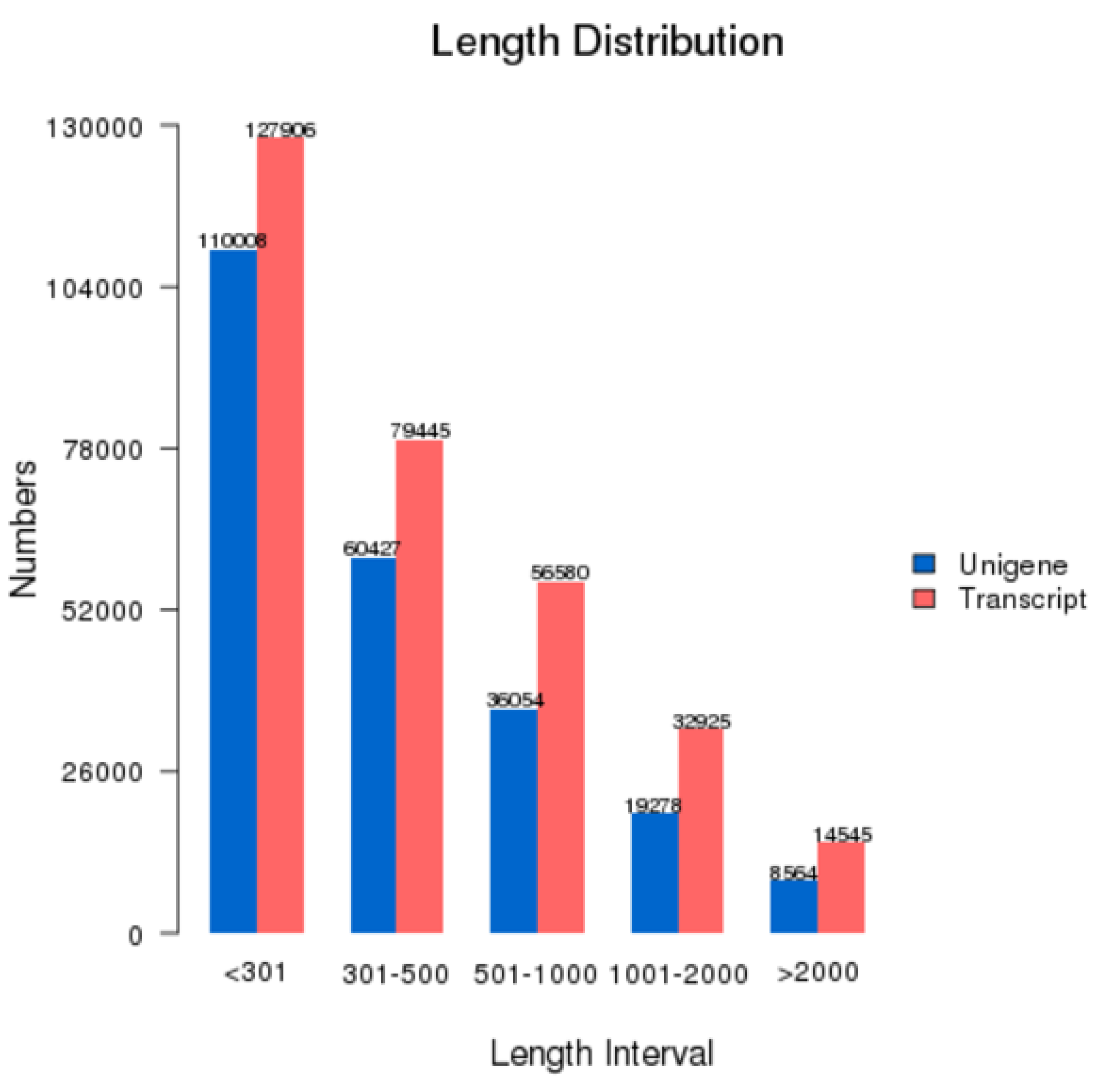

Supplement: S1 Fig — (TIF) [file pone.0212514.s001.tif]

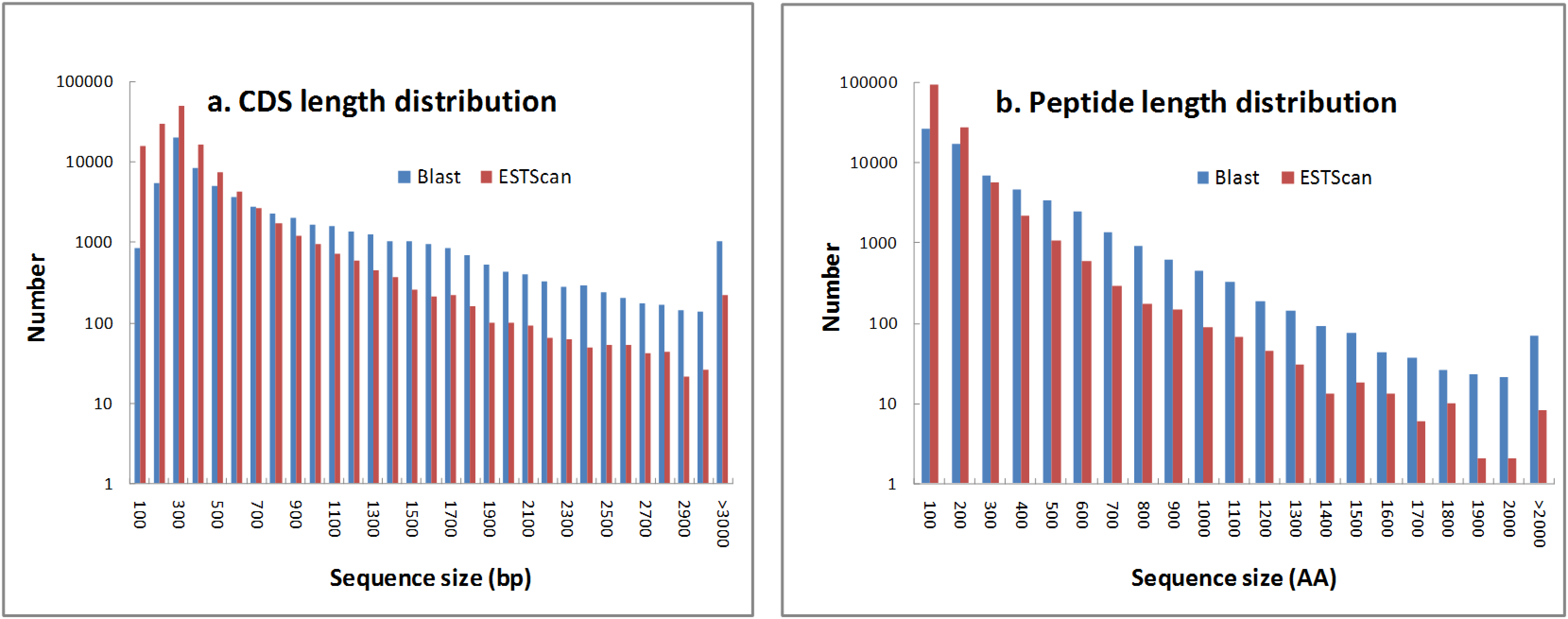

Supplement: S2 Fig — (TIF) [file pone.0212514.s002.tif]

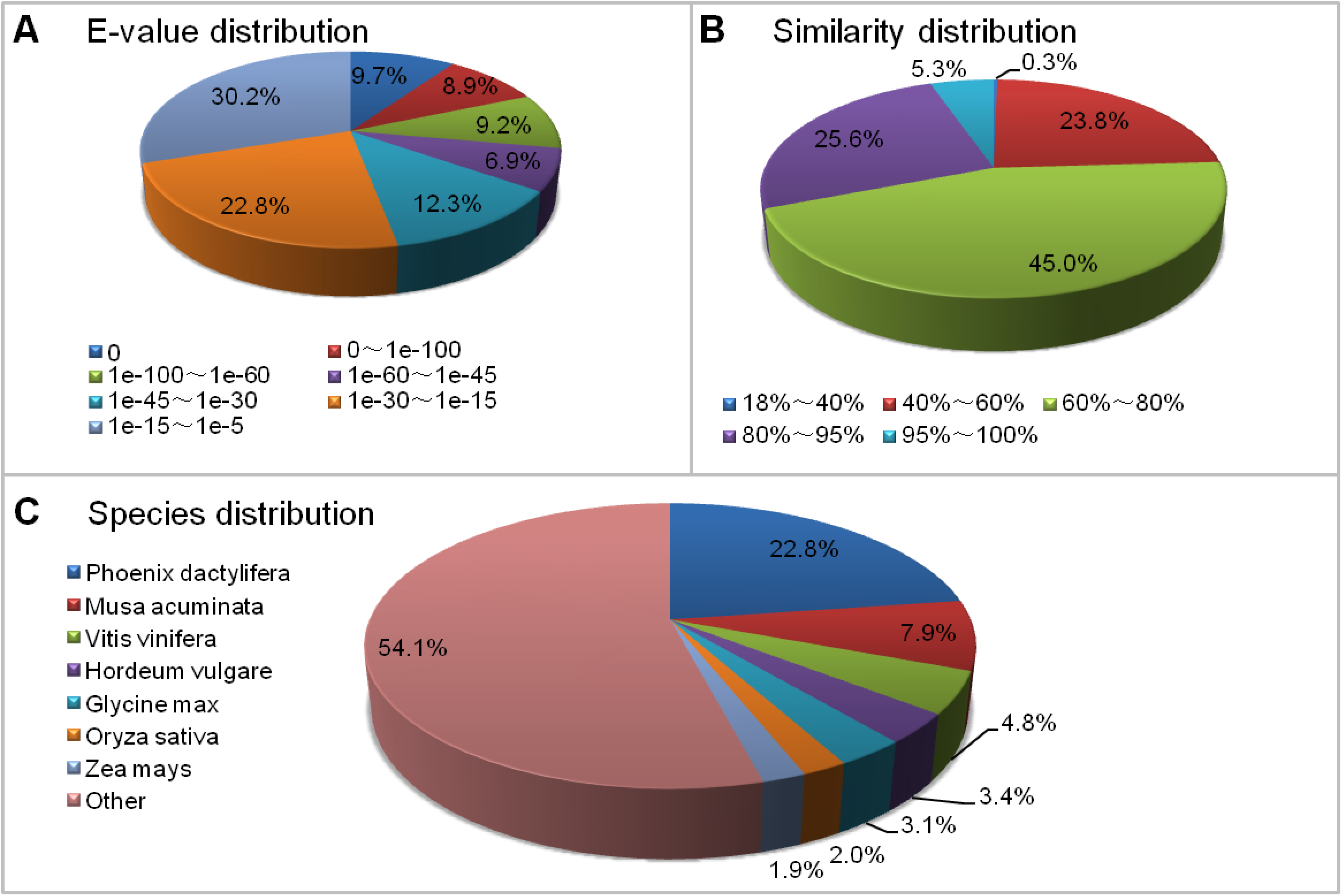

Supplement: S3 Fig — (TIF) [file pone.0212514.s003.tif]

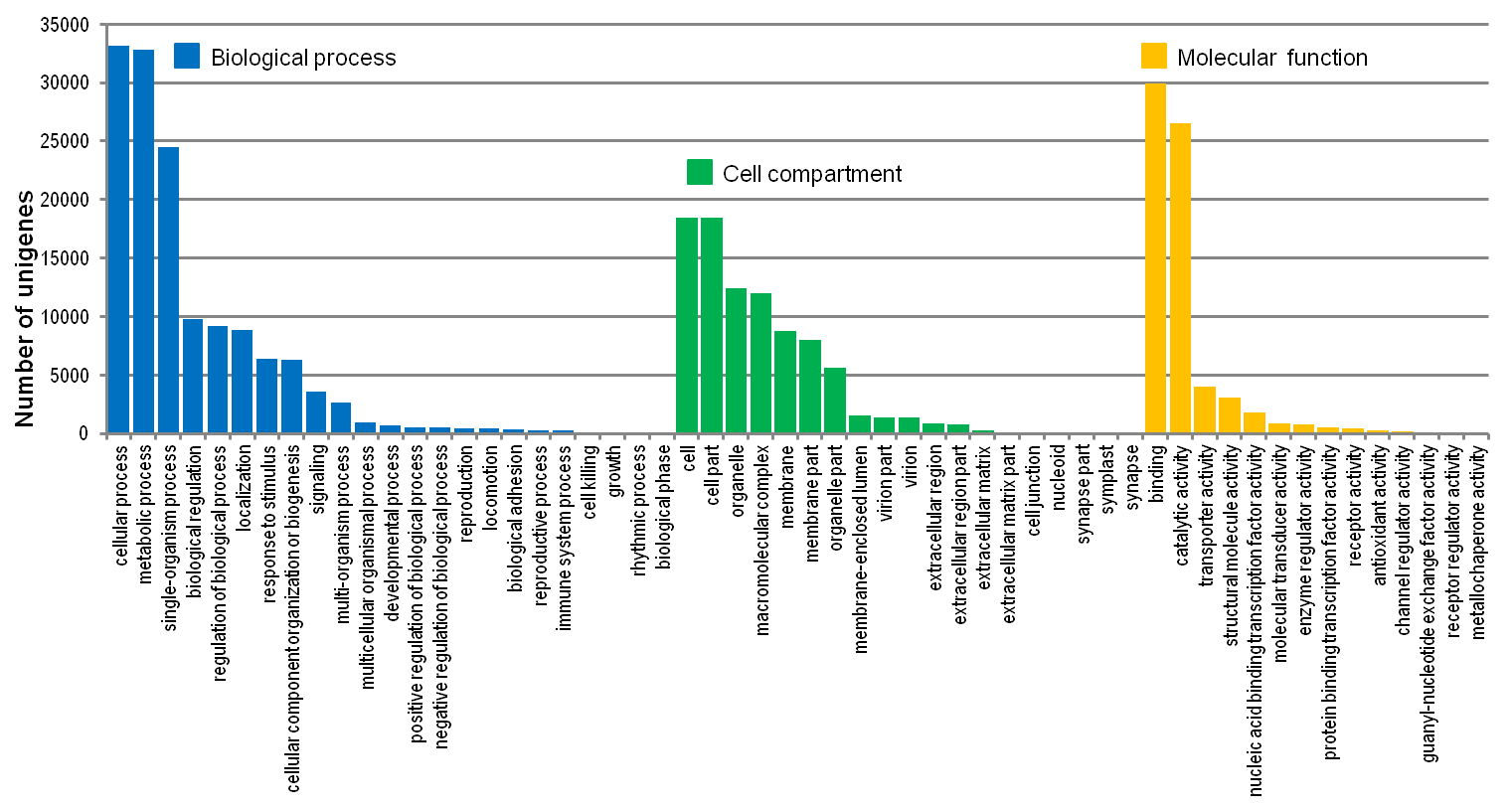

Supplement: S5 Fig — (TIF) [file pone.0212514.s005.tif]

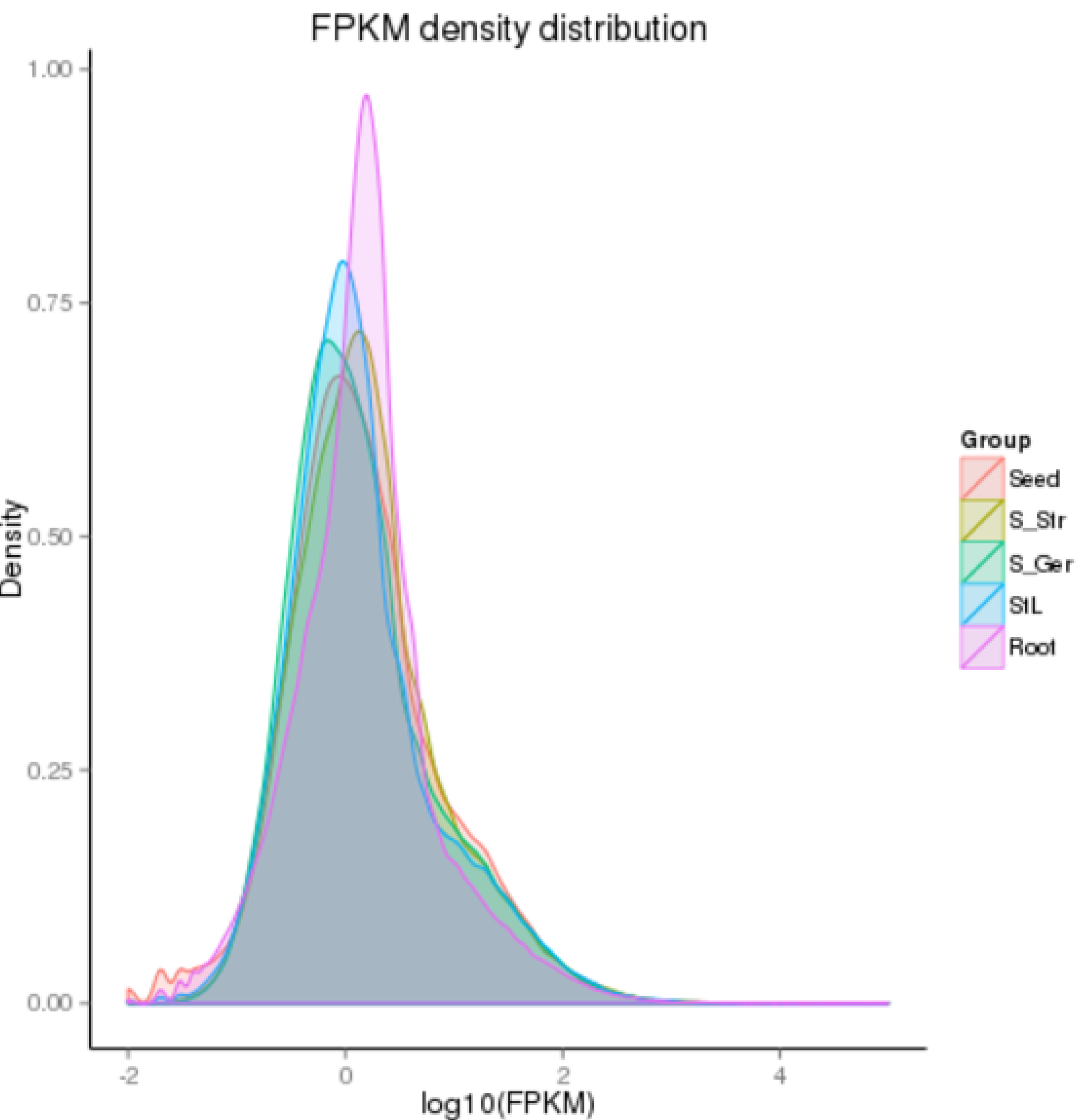

Supplement: S6 Fig — The x-axis and y-axis represent the gene log10 (FRKM) values and densities, respectively. Seed, S_Str, S_Ger, StL, and Root (here and afterward) correspond to mature seed (Fig 1A), stratified seed (Fig 1B), germinating seed (Fig 1C), stem and leaf, and root samples, respectively. (TIF) [file pone.0212514.s006.tif]
