# Supplementary material for: Comparative transcriptomic analysis reveals genes regulating the germination of morphophysiologically dormant Paris polyphylla seeds during a warm stratification
Source: PLoS One. 2019 Feb 21;14(2):e0212514. doi: 10.1371/journal.pone.0212514 (PMC6383930; doi:10.1371/journal.pone.0212514)
Supplement: S4 Fig — (PDF) [file pone.0212514.s004.pdf]

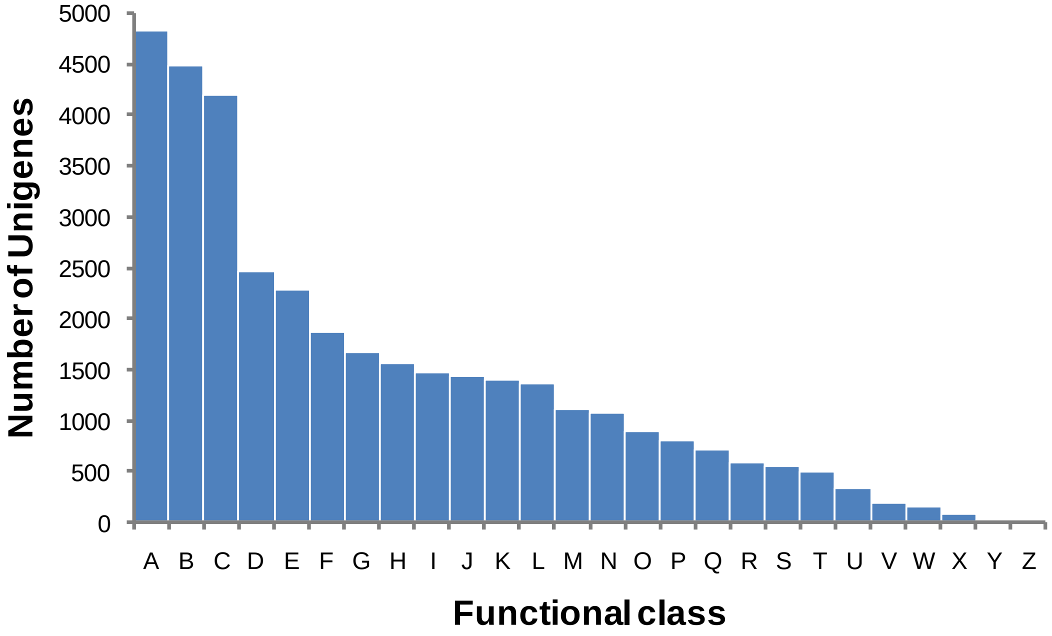

S4 Fig. Classification of *P. polyphylla* unigenes based on the COG database.

A. General function prediction only; **B.** Translation, ribosomal structure and biogenesis;

C. Posttranslational modification, protein turnover, chaperones; **D.** Signal transduction mechanisms;

E. Energy production and conversion; **F.** Intracellular trafficking, secretion, and vesicular transport;

G. Amino acid transport and metabolism; **H.** Lipid transport and metabolism; **I.** RNA processing and modification; **J.** Transcription; **K.** Carbohydrate transport and metabolism ; **L.** Secondary metabolites biosynthesis, transport and catabolism; **M.** Function unknown; **N.** Cytoskeleton; **O.** Inorganic ion transport and metabolism; **P.** Cell cycle control, cell division, chromosome partitioning; **Q.** Replication, recombination and repair; **R.** Chromatin structure and dynamics; **S.** Coenzyme transport and metabolism; **T.** Nucleotide transport and metabolism; **U.** Cell wall/membrane/envelope biogenesis;

V. Defense mechanisms; **W.** Nuclear structure; **X.** Extracellular structures; **Y.**. Cell motility; **Z.** Unnamed protein
